# A chloroplast-localized pentatricopeptide repeat protein involved in RNA editing and splicing and its effects on chloroplast development in rice

**DOI:** 10.1186/s12870-022-03819-y

**Published:** 2022-09-13

**Authors:** Yanwei Wang, Zhimin Yang, Meng Zhang, Pengfei Ai

**Affiliations:** Collage of Food and Biology, Hebei University of Science and Technology, Shijiazhuang, 050018 Hebei China

**Keywords:** Rice, Chloroplast, PPR protein, RNA editing, RNA splicing

## Abstract

**Background:**

The chloroplast is the organelle responsible for photosynthesis in higher plants. The generation of functional chloroplasts depends on the precise coordination of gene expression in the nucleus and chloroplasts and is essential for the development of plants. However, little is known about nuclear-plastid regulatory mechanisms at the early stage of chloroplast generation in rice.

**Results:**

In this study, we identified a rice (*Oryza sativa*) mutant that exhibited albino and seedling-lethal phenotypes and named it *ssa1(seedling stage albino1).* Transmission electron microscopy (TEM) analysis indicated that the chloroplasts of *ssa1* did not have organized thylakoid lamellae and that the chloroplast structure was destroyed. Genetic analysis revealed that the albino phenotypes of *ssa1* were controlled by a pair of recessive nuclear genes. Map-based cloning experiments found that *SSA1* encoded a pentapeptide repeat (PPR) protein that was allelic to *OSOTP51,*which was previously reported to participate in Photosystem I (PSI) assembly. The albino phenotype was reversed to the wild type (WT) phenotype when the normal *SSA1* sequence was expressed in *ssa1* under the drive of the actin promoter. Knockout experiments further created mutants *ssa1–2/1–9,* which had a phenotype similar to that of *ssa1*. SSA1 consisted of 7 pentatricopeptide repeat domains and two C-terminal LAGLIDADG tandem sequence motifs and was located in the chloroplast. GUS staining and qRT–PCR analysis showed that *SSA1* was mainly expressed in young leaves and stems. In the *ssa1* mutants, plastid genes transcribed by plastid-encoded RNA polymerase decreased, while those transcribed by nuclear-encoded RNA polymerase increased at the mRNA level. Loss-of-function SSA1 destroys RNA editing of *ndhB-737* and intron splicing of *atpF* and *ycf3–2* in the plastid genome. Yeast two-hybrid and BiFC assays revealed that SSA1 physically interacted with two new RNA editing partners, OsMORF8 and OsTRXz, which have potential functions in RNA editing and chloroplast biogenesis.

**Conclusions:**

Rice *SSA1* encodes a pentatricopeptide repeat protein, which is targeted to the chloroplast. SSA1 regulates early chloroplast development and plays a critical role in RNA editing and intron splicing in rice. These data will facilitate efforts to further elucidate the molecular mechanism of chloroplast biogenesis.

**Supplementary Information:**

The online version contains supplementary material available at 10.1186/s12870-022-03819-y.

## Background

Photosynthesis is one of the largest biochemical reactions occurring on earth and plays an important role in maintaining energy metabolism and carbon skeleton synthesis in living organisms. As a semiautonomous organelle, chloroplasts are not only important sites of photosynthesis but are also responsible for the biosynthesis and storage of metabolites in higher plants [41, 44]. Chloroplasts develop from proplastids, and their development can be divided into three periods: plastid genome replication, construction of the translation system and establishment of the chloroplast light harvesting system [25]. The formation of chloroplasts is cocontrolled by nuclear coding polymerase (NEP) and plastid coding polymerase (PEP) [73]. NEP is a functional protein that is synthesized by the nucleus and transported into the chloroplast to perform its function. It is mainly responsible for gene transcription of the core subunit of PEP, partial ribosomal proteins, and other “housekeeping” proteins in plastids [43]. PEP is a large complex protein containing many dynamic peripheral factors and is responsible for the transcription and synthesis of some photosynthesis-related proteins in chloroplasts as well as proteins related to the growth and development of the chloroplasts themselves [73]. Loss of function of proteins involved in PEP and NEP could generate the plant albino phenotype [31, 43, 48, 71]. In Arabidopsis, the deficiency of *thioredoxin z* (*TRXz*) leads to yellow wilting leaves when grown on agar plates and subsequent death in the seedling stage. *TRXz* encodes chloroplast thioredoxin, which can interact with two fructokinase-like proteins, FLN1 and FLN2, by conserved Cys residues [1]. The superoxide dismutase gene *ALM1* can also interact with PEP complex *OsTRXz* and regulate chloroplast development by affecting the content and activity of reactive oxygen species in rice [62–64]. Mutation of the *AL3 gene,* which encodes a plastid caseinolytic protease, causes albino leaves and seedling death in rice [34].

RNA editing was first observed in protozoan trypanosomabrucei mitochondria in 1986 [3]. It was discovered that the mRNA precursors of glutamate receptors underwent a new posttranscriptional modification and the concept of “RNA editing” was proposed in the early 1990s [47]. To date, RNA editing has been widely found in various biological organelles, including mitochondria, chloroplasts and the nucleus [46]. In higher plants, RNA editing usually occurs during the posttranscriptional regulation of mitochondria or the chloroplast genome. Through RNA editing, cytosine (nucleotide C) in mRNA can be transformed into uracil (nucleotide U), thus changing the genetic information of plants [17]. Although RNA editing was first discovered 30 years ago, the editing complex has only recently been revealed. To date, scientists have reported a variety of protein factors associated with RNA editing, including MORF (Multiple Organellar RNA Editing Factors), OZ (Organelle Zinc Fingers), ORRM (Other RRM proteins), PPO1 (Protoporphyrinogen IX Oxidase 1), and PPR (Pentatricopeptide Repeat) proteins [45, 46, 74, 75]. In *Arabidopsis thaliana*, the MORF1 protein is located in the mitochondria, and its functional loss can cause abnormal RNA editing at approximately 40 sites with in mitochondria with reduced fertility and seed setting rates [52]. Co-IP screening and mass spectrometry analysis found that OZ1 could interact with ORRM1 and regulate RNA editing at multiple sites in Arabidopsis chloroplasts. Together they affect chloroplast development [50]. In addition, by introducing a new chloroplast RNA editing site, *PsbF-26* in tobacco, analysis found that, there was no RNA editing factor capable of editing this site in tobacco, but *PsbF-26* could be edited by introducing the PPR protein LPA66 from Arabidopsis. This means that the PPR protein has the ability to edit RNA [35]. Among these RNA editing factors, the PPR protein is a common key factor that mediates the specific recognition of RNA editing sites [19]. To date, 24 RNA editing sites have been found in the chloroplast genome of rice [9], but the relationship between editing factors and recognition sites has rarely been studied. Meanwhile, the function of many RNA editing-related genes need to be further explored.

The PPR protein is a plant-specific protein characterized by repeated sequences of multiple PPR motifs (approximately 35 amino acid repeats). The PPR family, which contains 450 proteins in Arabidopsis and 491 proteins in rice, is one of the largest gene families in plants [6]. According to their protein sequence structure, PPR proteins can be divided into two subgroups, P type and PLS type. Protein members of the P-type subfamily usually have a standard 35 amino acid repeat motif but do not contain other sequences. Their functions are mainly manifested as the abilities to perform cell organelle RNA splicing [4, 67, 70], maintain RNA stability [32, 59] and initiate the protein translation process [13, 27, 32, 59]. PLS subfamily proteins are composed of standard P motifs, longer L motifs and shorter S motifs. The members of this subfamily are mainly responsible for regulating the editing process of RNA from C to U after transcription of the organelle genome [23, 38, 56]. Most PPR proteins play important biological functions in chloroplasts or mitochondria and are involved in multiple biological processes of plant growth and development, such as embryogenesis [45, 51, 59], seed development [30], chloroplast development [7, 39, 49], cytoplasmic male sterility [18, 76, 78], and retrograde signalling [24, 26]. Several PPR proteins have been reported in the chloroplast of rice. After *WSL5* gene mutation, intron splicing of the chloroplast genes *rps12* and *rpl2* could not be carried out normally. Meanwhile, RNA editing at some sites of *atpA* and *rpl2* was abnormal, leading to defective chloroplast development and plant albinism under low temperature in rice [33]. DUA1 is involved in RNA editing of the chloroplast gene *rps8* and is an essential regulatory factor in chloroplast development under low temperature [10]. OsPPR4 regulates chloroplast development by affecting intron splicing of *atpF*, *ndhA*, *rpl2* and *rps12* as well as RNA editing of *ndhA* in rice [2]. OsPGL1 is a dual-localized protein in both chloroplasts and mitochondria, and its functional defects can lead to abnormal RNA editing of *ndhD* and *ccmFc*, thus controlling plant development and chlorophyll content [69]. OsPPR6 can control RNA editing of the chloroplast gene *ndhB* and RNA splicing of *ycf3*. The *osppr6* mutant becomes albino lethal at the seedling stage [54]. After the *OsPPR16* gene was knocked out, the plant showed abnormal chloroplast development and decreased chlorophyll content at the seedling stage and then returned to the WT phenotype after the five-leaf stage [16]. OsATP4 also regulates chloroplast development at low temperatures, similar to DUA1 in rice [76, 78]. Even though PPR proteins are crucial for the growth and development of plants, we know very little about their biological functions. Subsequent studies on key PPR proteins will help to further expand the understanding of genetic pathways regulating plant growth and development and promote crop molecular breeding in rice.

Here, we isolated a new PPR protein, SSA1, which is allelic to the *OsOTP51*gene*,* that plays roles in PSI assembly and in regulating chloroplast development in rice [72]. The results showed that *SSA1* interacts with *OsMORF8* and *OsTRXz* in vitro and in vivo. Mutations in *SSA1* affect NEP- and PEP-related gene expression and RNA processing. Our study reveals a new mechanism by which the PPR-MORF-TRXz module regulates chloroplast gene expression and chloroplast development in rice.

## Results

### Phenotypic and genetic characterization of *ssa1*

**Fig. 1:**
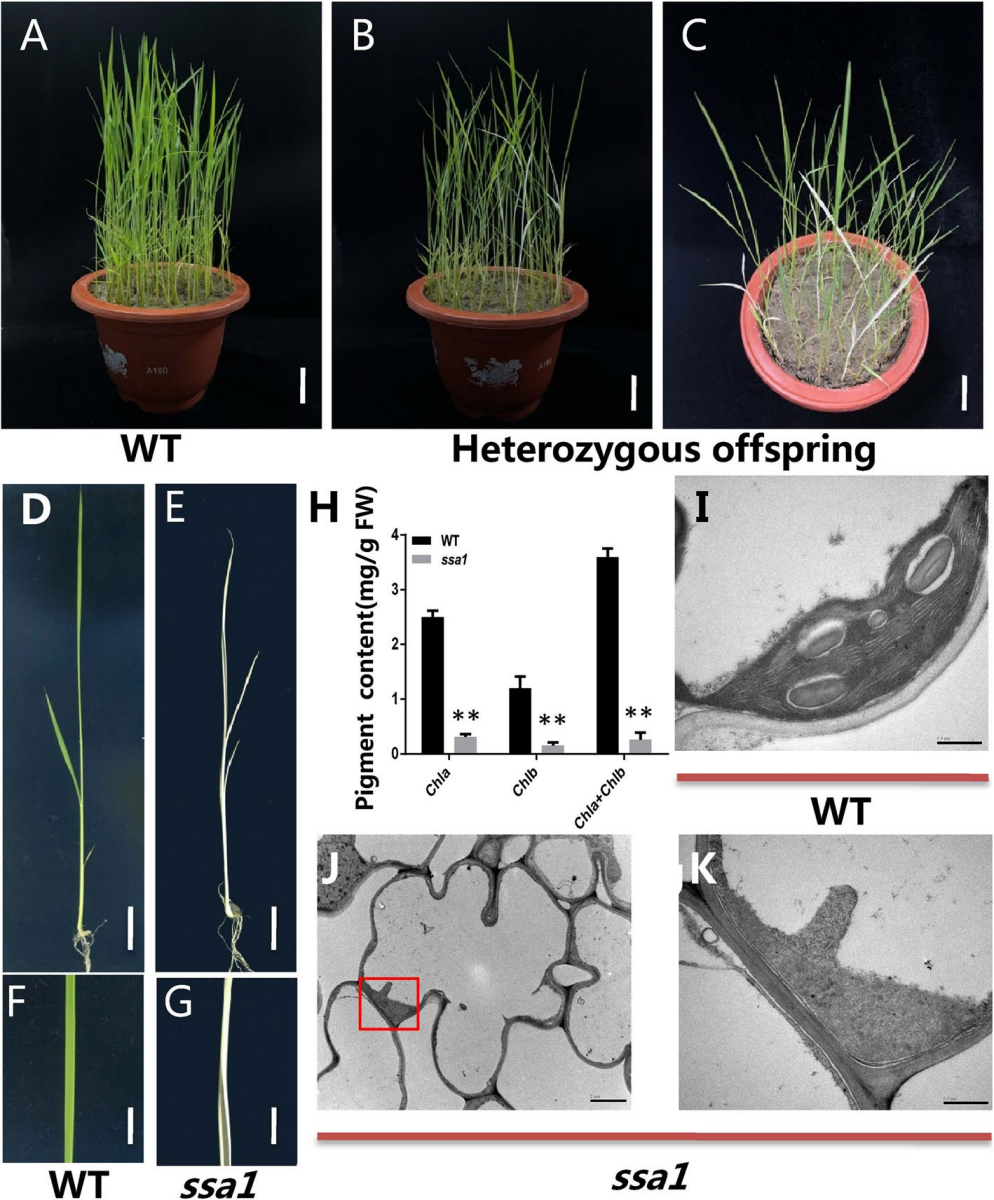
The phenotype analysis of WT and *ssa1.* **A** Phenotypes of WT. **B** The offspring isolated from a heterozygous plant (Side view observation). **C** Offspring isolated from a heterozygous plant (overlooking observation). **A, B, C** Bar = 5 cm. **D** Phenotypes of WT. **E** Phenotypes of *ssa1*. **D, E** Bar = 2 cm. **F** The leaf phenotype of WT. **G** The leaf phenotype of mutant *ssa1*. **F, G** Bar = 1 cm. **H** Chlorophyll content in WT and mutant *ssa1.* Chl a, chlorophyll a; Chl b, chlorophyll b; FW, fresh weight. SD was calculated from 5 independent plant. (**P < 0.01,Student’s t test). **I** Chloroplast ultrastructure in WT. **J** Chloroplast ultrastructure in *ssa1.* **K** the amplification of red rectangle in (**J**). **I, K** Bar = 0.5 μm, **J** Bar = 1 μm

To identify the components that play a role in chloroplast development, we screened our mutant library and searched rice plants for leaves that were different from normal rice leaves. We obtained genetic material *ssa1* mutant*,* which shows albinism when grown in the field from Nipponbare, a japonica rice variety. The characterization of this mutant was analysed in further detail and the data showed that the mutation of *SSA1* resulted in a slightly shorter plant height (Fig. 1A-C) and reduced chlorophyll content (Fig. 1H). The albino phenotype appeared in the whole seedling after seed germination in *ssa1*, but the growth was completely normal before the four-leaf stage (Fig. 1A-G). Then, the *ssa1* plant withered and died at the seedling stage after the four-leaf stage. Transmission electron microscopy (TEM) was used to observe the cytological structure of leaves. The chloroplast structure was almost unaffected in WT, while the chloroplasts collapsed and shrank in *ssa1* (Fig. 1I-K). The above results indicated that the albino phenotype in *ssa1* was perhaps caused by damage to chloroplast structure.

### Map-based cloning of *SSA1*

**Fig. 2:**
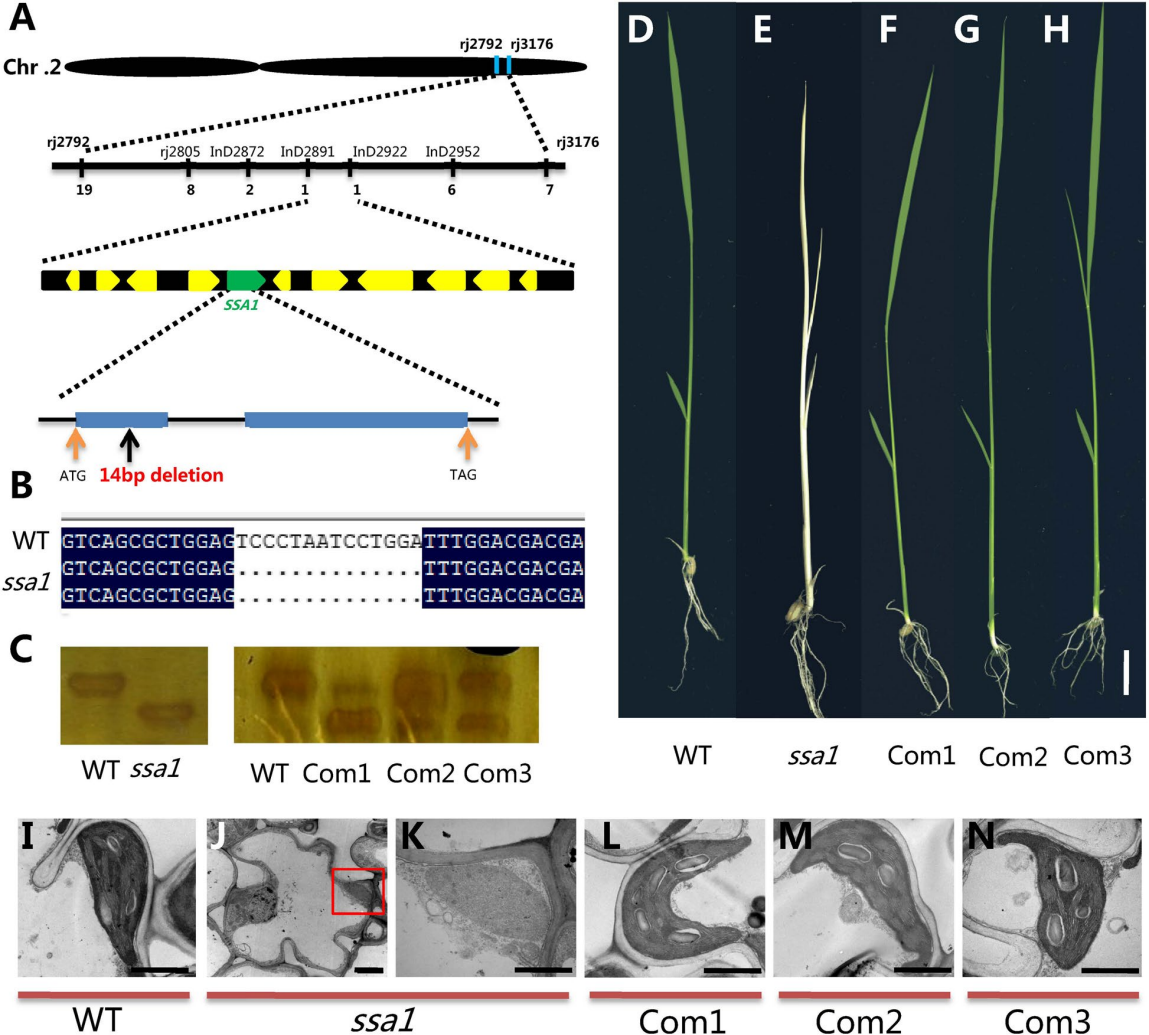
Map-based cloning of the *ssa1* locus and complementary validation. **A** The *ssa1* locus was fine mapped to a interval between markers InD2891 and InD2922 on chromosome 2 (Chr. 2). The yellow and green arrowhead represent putative genes in the region. The blue rectangle represents exon and the black line represents the intron of target gene. There is a 14 bp deletion on the first exon of the *SSA1*. **B** The mutant target gene was sequenced and compared with the WT. **C** Specific molecular markers were designed on the mutation sites flanking on the exons. The molecular weight of *ssa1* mutant plants decreased. After molecular identification, double bands were found in transgenic complementary plants. **D** WT plant appearance phenotype. **E** *ssa1* mutant plant appearance phenotype. **F-H** Complementary line plant appearance phenotype. **D-H** Bar = 5 cm. **I-N** Chloroplast ultrastructure in WT (**I**), *ssa1* (**J-K**) and complementary transgenic line (**L-M**). K represent the amplification of red rectangle in J. Bar = 1 μm (**I-N**)

To discover the target gene that controls the phenotype of *ssa1*, we analysed its genetic characteristics and constructed a genetic mapping population by means of a cross between the *ssa1* and WT indica cultivars (Dular). The cross experiment were processed between the *ssa1* heterozygotic plants and Dular. The heterozygous F1 generation was selected for subsequent experiments. Genetic statistical analysis revealed that the albino phenotype in *ssa1* was controlled by a recessive nuclear gene (F2 segregation: 309 plants normal green; 98 plants albinism; Chi^2^_3:1_ = 0.357, *P* = 0.51). Initially, *SSA1* was mapped at the physical position of 28 M on the short arm of chromosome 2. Then, new molecular markers were designed to narrow the locus interval. Finally, *SSA1* was located within the range of approximately 100 kb between the markers InD2891 and InD2922 (Fig. 2A). Further sequencing analysis revealed a fourteen base deletion in the gene *LOC_Os02g47360* in *ssa1,* which resulted in frameshift mutations of the encoded amino acids and caused premature termination of protein translation (Fig. 2B, S1A). The truncated *ssa1* protein had an obviously changed 3D structure (https://zhanggroup.org/I-TASSER/) (Fig. S1B). These results indicated that *LOC_Os02g47360* was perhaps the candidate gene of *ssa1*.

### Phenotypic complementation and reappearance of *ssa1*

**Fig. 3:**
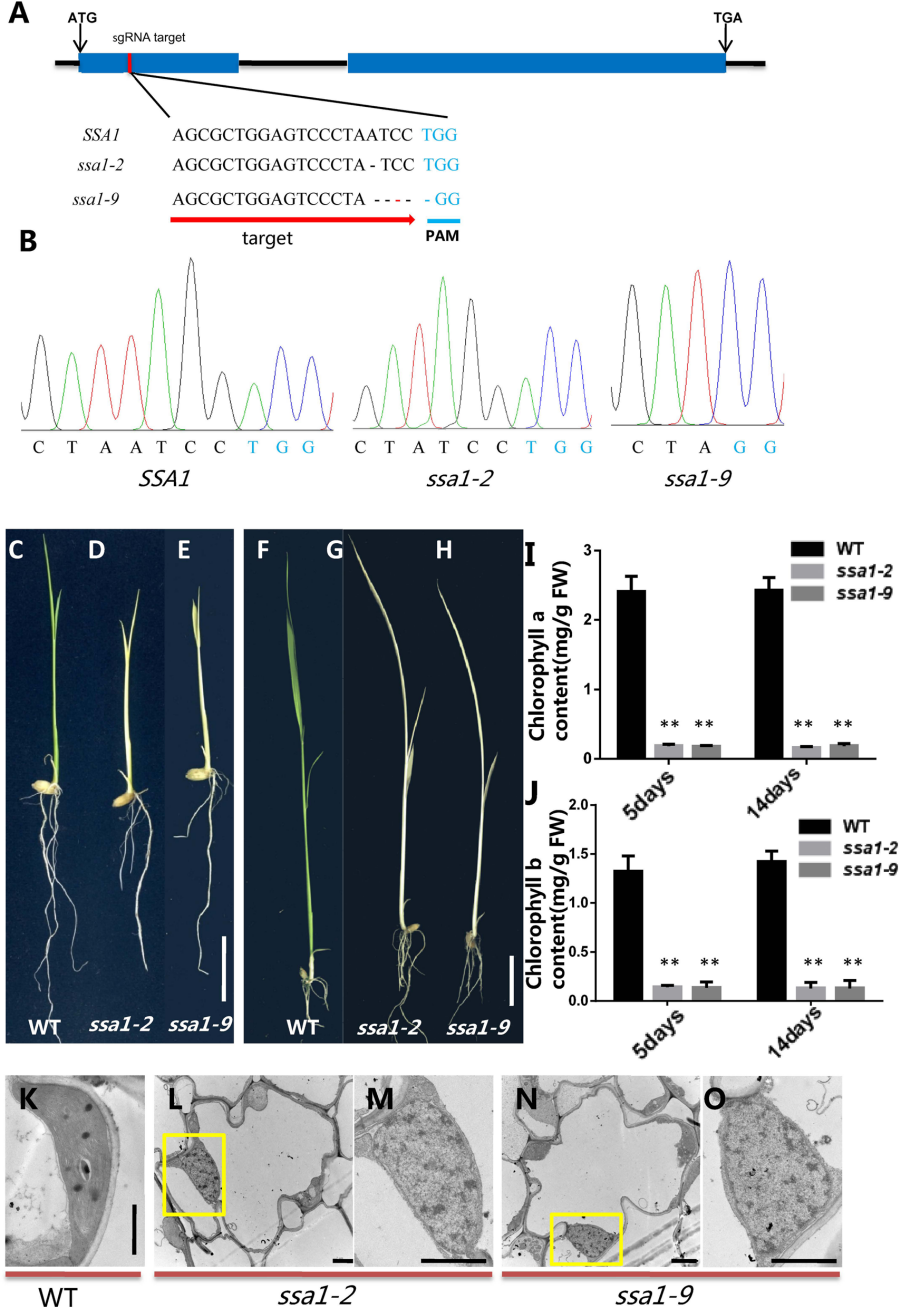
CRISPR/Cas9-targeted mutagenesis of *SSA1.* **A** Selection of knockout target sites and sequence alignment between WT and transgenic plants. **B** WT and knockout transgenic plants sequencing identification. **C-E** Phenotypic of WT(**C**), knockout plants *ssa1–2* (**D**) and knockout plants *ssa1–9* (**E**) in 7 days after planting. **F-H** Phenotypic of WT (**F**), knockout plants *ssa1–2* (**G**) and knockout plants *ssa1–9*(**H**) in 14 days after planting. **I** Chlorophyll a contents of 7 day and 14 day old WT and knockout seedlings. SD was calculated from 5 independent plant. (**P < 0.01,Student’s t test). **J** Chlorophyll b contents of 7 day and 14 day old WT and knockout seedlings. SD was calculated from 5 independent plant. (**P < 0.01, Student’s t test). **K** Chloroplast ultrastructure in WT. **L** Chloroplast ultrastructure in knockout line *ssa1–2.* **M** Enlarged view of yellow rectangle in (**L**). **N** Chloroplast ultrastructure in knockout line *ssa1–9*. **O** Enlarged view of yellow rectangle in (**N**). Bar = 1 μm in (**K-O**)

To verify the result that *LOC_Os02g47360* is responsible for the mutant phenotype, the full-length CDS of the WT *SSA1* gene was fused with pCambia 2300 and expressed by the actin promoter (Fig. S2A). Transgenic plants were identified by PCR amplification, and three positive plants (Com1, Com2 and Com3) with independent transformation events were selected for later phenotypic study (Fig. 2C). All positive complementary line plants developed normal green leaves at seedling stages (Fig. 2D-H) and were restored to WT chlorophyll levels (Fig. S2B). The structure of chloroplasts was also detected by TEM, and the results showed that all chloroplast structures in the complementary line were normal and consistent with those in WT (Fig. 2I-N). To further confirm the result that the phenotype of *ssa1* was caused by the functional loss of *LOC_Os02g47360*, the CRISPR–Cas9 system was used to construct a knockout mutant of this gene. Molecular identification and phenotypic analysis were carried out on the knockout plants. Among the multiple knockout genetic lines obtained, we selected *ssa1–2* and *ssa1–9* as two representative lines for subsequent experimental verification, which had one base missing and five bases missing, respectively (Fig. 3A-B). Sequence analysis showed that both of these transgenic lines caused the premature termination of protein translation (Fig. S3). Phenotypic observation showed that the transgenic lines *ssa1–2* and *ssa1–9* showed albinism at the seedling stage with reduced chlorophyll content (Fig. 3C-J). Meanwhile, the TEM results showed that there were malformed immature chloroplasts in the CRISPR–Cas9 lines *ssa1–2* and *ssa1–9*, similar to that in *ssa1* (Fig. 3K-O). Collectively, the above results indicated that the *LOC_Os02g47360* gene was responsible for the *ssa1* albino and chloroplast defect phenotypes.

### *SSA1* encodes a P-type PPR protein

Sequence analysis showed that the full-length cDNA sequence of *SSA1* was 2373 bp in the NCBI database (https://www.ncbi.nlm.nih.gov). The SSA1 protein has two exons with 790 amino acids. The mutant site was located in the first exon 184 base pairs from the start site in *ssa1* (Fig. 2A). SSA1 contains 7 canonical PPR motifs according to prediction by TPRpred (https://toolkit.tuebingen.mpg.de/tools/tprpred). There are two LAGLIDADG sequence motifs in the C-terminus (Fig. S4A). Multiple sequence alignment revealed that the amino acid sequence of SSA1 was highly conserved in plants and shared a high degree of sequence similarity with maize *GRMZM2G028605* (75.9% identity) and *Arabidopsis thaliana At2g15820* (43.7% identity) (Fig. S4B). To further identify the sequence homology relationship between SSA1 and other PPR proteins in rice, sequence comparison was performed. The PPR protein sequences that were more than 20% similar to SSA1 were selected for alignment. The results showed that no PPR protein with very high homology was found in rice (Fig. S5). We constructed a phylogenetic tree to further analyse the evolutionary origin and possible function of *SSA1*. The results indicated that the PPR protein encoded by *SSA1* has an orthologue in brachypodium (*Brachypodium distachyon*) and a paralogue in insetaria (*Setaria italica*), maize (*Zea mays*) and sorghum (*Sorghum bicolor*) (Fig. S6). Meanwhile, Together, these results indicated that *SSA1* encodes a new PPR protein that is allelic to *OsOTP51*.

### Expression pattern and subcellular localization of *SSA1*

**Fig. 4:**
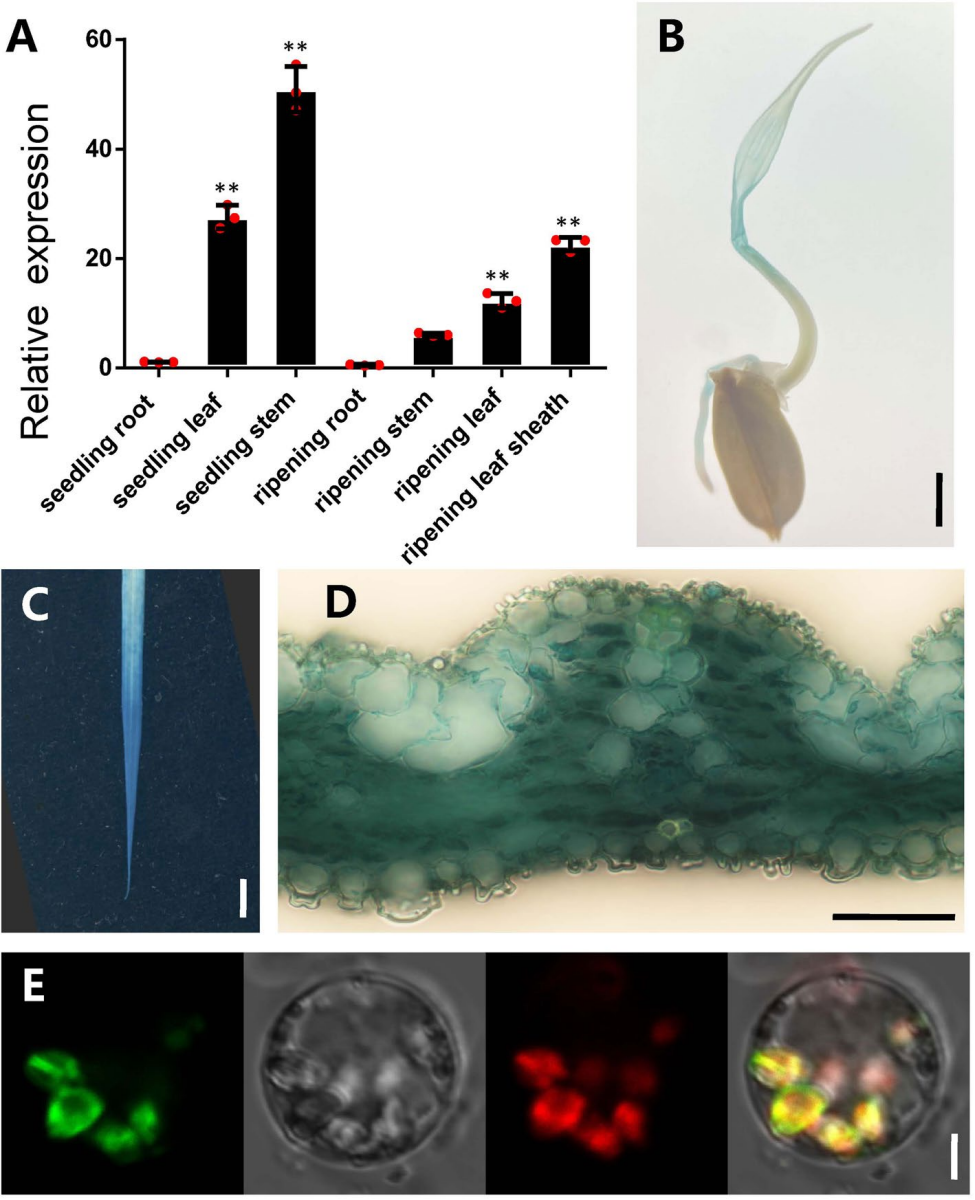
The expression and subcellular localization analysis of SSA1. **A** Transcript levels of *SSA1* in different tissues of WT seedlings. SD was calculated from 3 independent plant. **B** Histochemical staining showed that the pSSA1::GUS reporter gene was expressed in leaves in germinating seed. Bar = 0.5 cm (**C**) Histochemical staining showed that the pSSA1::GUS reporter gene was highly expressed in young leaves. Bar = 0.5 cm. **D** Histological section showed the GUS staining in young leaf. Bar = 50 μm. **E** Subcellular localization of SSA1 protein. Green, red and yellow fluorescence show GFP, chloroplast autofluorescence, and the merged fluorescence, respectively. Bar = 5 μm

By searching the Rice eFP Browser (http://bar.utoronto.ca/efprice/cgi-bin/efpWeb.cgi), we found that *SSA1* was expressed mainly in leaves (Fig. S7A). The database RiceXPro (https://ricexpro.dna.affrc.go.jp) also showed leaf expression characteristics of *SSA1* (Fig. S7B). To further confirm these data, we conducted qRT–PCR to detect the expression of *SSA1* in roots, leaves, and stems at the seedling stage and roots, leaves, stems and leaf sheaths at the mature stage. The results showed that the expression of *SSA1* was highest in leaves and stems (Fig. 4A). *Promoter*_*SSA1*_-GUS fused vectors were also constructed and used to infect rice callus mediated by *Agrobacterium tumefaciens*. In positive transgenic plants, the whole leaves showed strong staining at the seedling stage, similar to the qRT–PCR results (Fig. 4B-D). This experimental evidence indicated that *SSA1* was mainly expressed in young leaves. Most PPR proteins are thought to be located in chloroplasts or mitochondria. In our study, SSA1 contained a targeted chloroplast signal peptide when predicted by the TARGETP website (www.cbs.dtu.dk/services/TargetP/) (Fig. S4A). To further confirm whether the SSA1 protein localized to the chloroplast, the fused vector containing the full-length SSA1 coding sequence with the 35S promoter was constructed and transformed into rice protoplasts with the help of PEG solution. Microscopic observations showed that fluorescent proteins expressed by SSA1 fused with GFP and merged with chloroplast autofluorescence (Fig. 4E). This means that the SSA1 protein localized in chloroplasts and played a role in chloroplast tissue. Together with chloroplast localization, the *ssa1* phenotype supported the notion that SSA1 mediated chloroplast development in rice seedlings.

### The expression of photosynthesis-related genes is Downregulated in *ssa1*

**Fig. 5:**
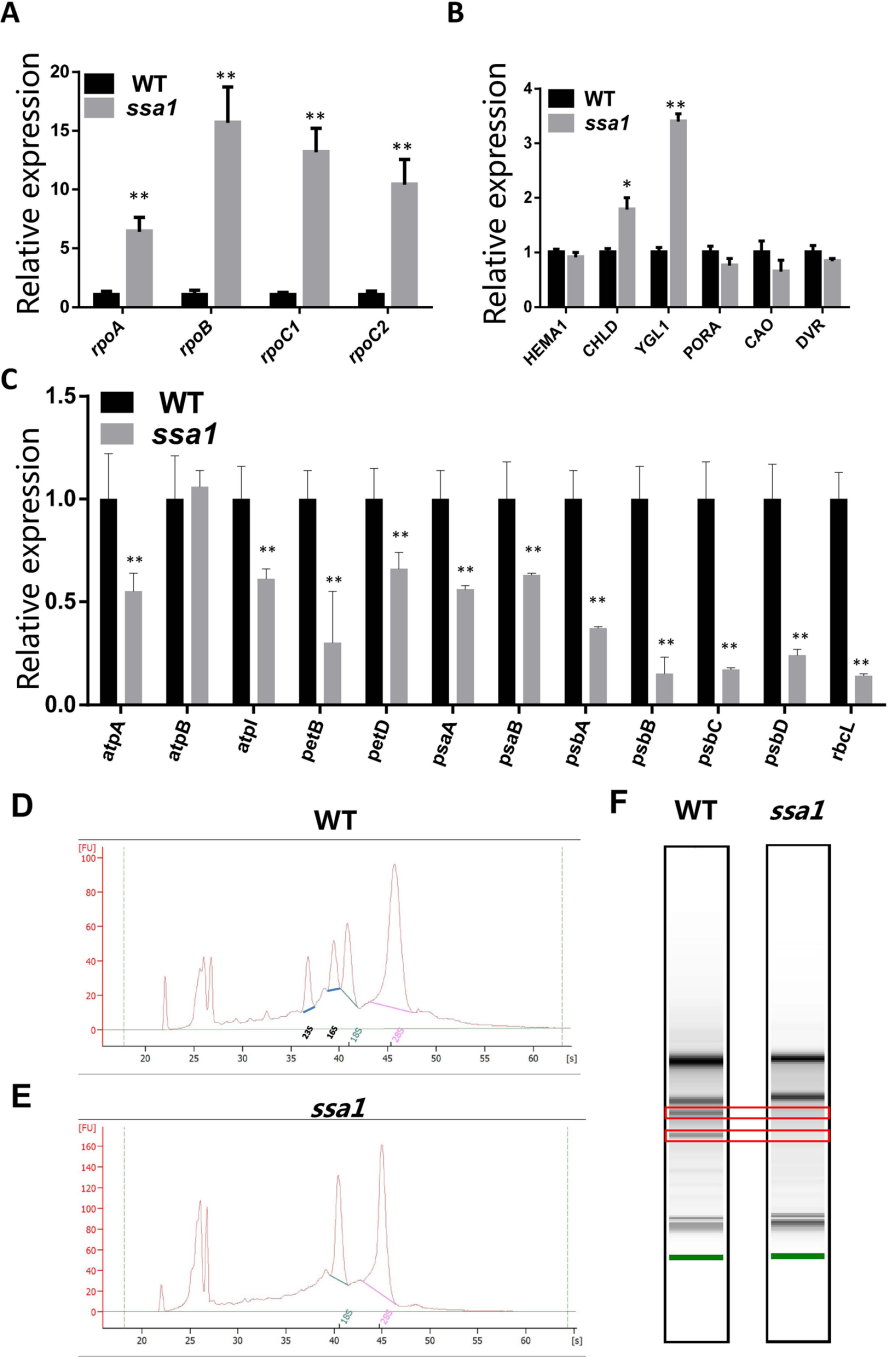
Accumulation of transcripts of chloroplast-associated genes in WT and *ssa1* seedlings. **A** qRT-PCR analysis of relative expression levels of plastidic PEP core genes in WT and ssa1 mutant at the third-leaf stage. SD was calculated from 3 independent plant. (**P < 0.01, Student’s t test). **B** qRT-PCR analysis of relative expression levels of chlorophyll synthesis genes in WT and *ssa1* mutant at the third-leaf stage. SD was calculated from 3 independent plant. (**P < 0.01, Student’s t test). **C** qRT-PCR analysis of relative expression levels of photosynthesis and chloroplast development genes in WT and *ssa1* mutant at the third-leaf stage. SD was calculated from 3 independent plant. (**P < 0.01, Student’s t test). **D-F** rRNA analysis using an Agilent 2100. RNA was isolated from WT seedlings (**D, F**, left lane) and *ssa1* seedlings (**E, F**, right lane). The bands in the red box represent 23S and 16S rRNA that missing in *ssa1*

Chloroplast development and functional genesis depend on the genes encoded by both the chloroplast and nuclear genomes. Chloroplast transcription machinery is regulated in a coordinated way by both NEPs and PEPs. Given that the phenotype of *ssa1* mainly occurs in chloroplasts, we examined the expression levels of genes related to chloroplast biogenesis. It was confirmed that the stability of PEP polymerase plays an important role in chloroplast development. We used qRT–PCR to detect the transcription levels of plastid-encoded and photosynthesis-related genes of both the WT and the *ssa1* mutant. For four PEP core complexes, *rpoA*, *rpoB*, *rpoC1* and *rpoC2*,that were transcribed by NEP, the genes had higher expression in the *ssa1* mutant than in the WT plant (Fig. 5A). In regard to PEP-dependent genes, the expression levels of almost all of the photosynthesis-related genes were strikingly reduced in the mutants compared with those in the WT (Fig. 5C). These results suggested that the defect in *SSA1* impeded chloroplast development, possibly by disrupting the expression of genes involved in chloroplast biosynthesis. Moreover, we analysed the composition and content of rRNAs by using an Agilent 2100. The 23S rRNAs and 16S rRNAs were almost abolished in *ssa1* seedlings (Fig. 5D-F). These results implied that the plastid ribosomal RNA biogenesis system is impaired as a result of *SSA1* mutation. We also detected the expression of chlorophyll biosynthesis-related genes. Notably, *CAO* showed decreased expression, but *CHLD* and *YGL1* showed increased expression in *ssa1* (Fig. 5B). Other chlorophyll synthesis genes, including *HEMA*, *PORA* and *DVR,* did not significantly change (Fig. 5B). Considering the much lower chlorophyll content in *ssa1*, we speculated that *SSA1* was involved in chloroplast development and indirectly influenced chlorophyll synthesis. The transcript profiles of the PEP and NEP genes in *ssa1* were similar to those of *ptac2* and *clb19* mutants, which led to deficiency of the PEP complex [5, 43]. The above results imply that *ssa1* may be defective in PEP complex activity.

### *ssa1* is defective in the splicing of introns in the chloroplast genome

Previously, loss-of-function OSOTP51 was shown to affect intron splicing of a number of plastid genes, particularly *ycf3,*which encodes a protein involved in the assembly of the PSI complex [72]. To identify the effect of our mutant *ssa1* on RNA splicing, we carried out reverse transcription PCR (RT–PCR) with the specific primers listed in Table S1. All probable chloroplast transcripts were amplified by using primers flanking the introns, and the lengths of the amplified products of the WT and the *ssa1* mutant were compared. The results showed that *ycf3* was also not spliced at its second exon in *ssa1.* Meanwhile, the fully spliced *atpF* intron was also undetectable in *ssa1* (Fig. S8A-D). These results indicate that *SSA1* is involved in chloroplast intron splicing.

### SSA1 is required for RNA editing of the *ndhB* transcript

**Fig. 6:**
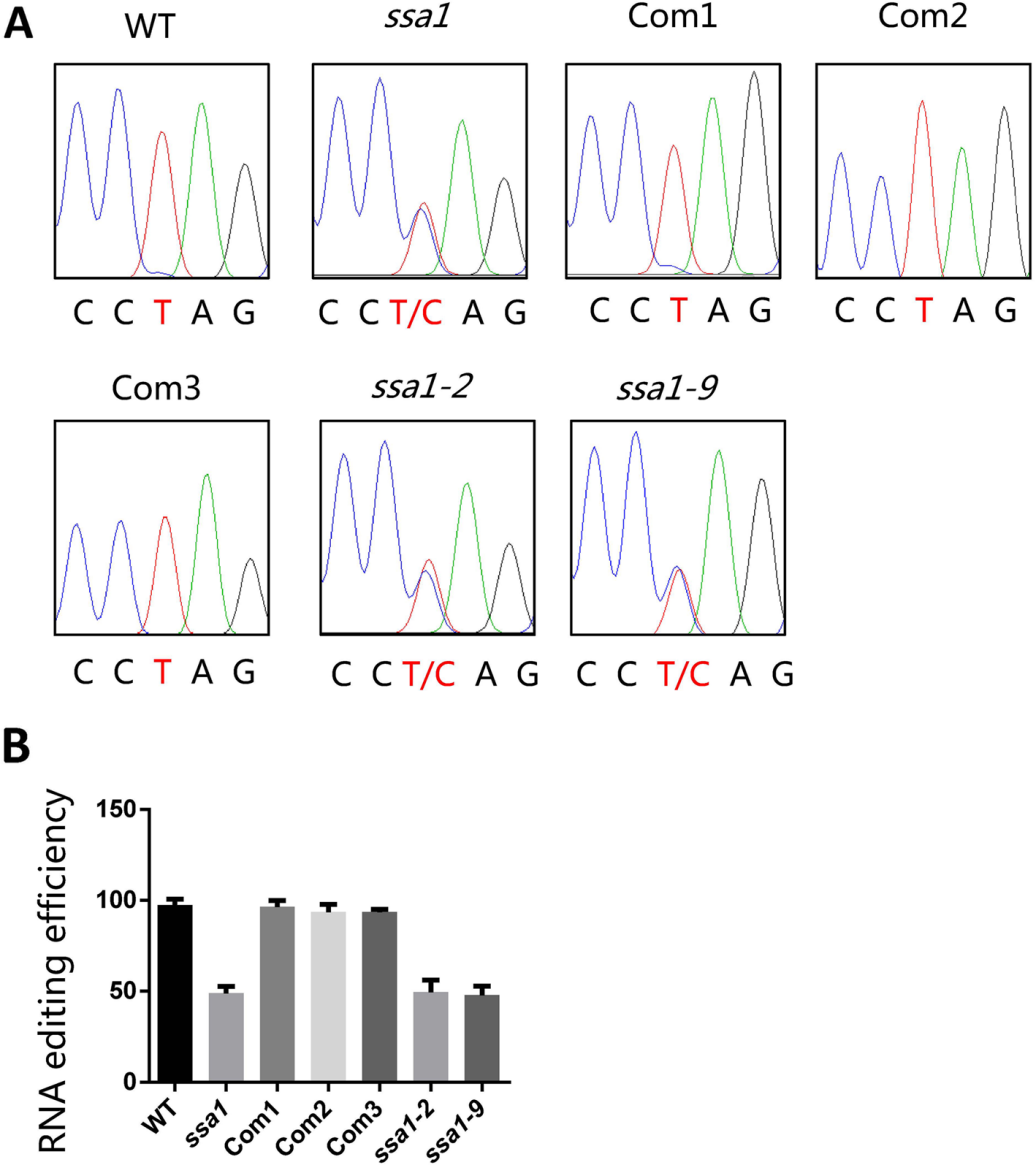
RNA Editing efficiencies of *ndhB* detection. **A** Sequencing chromatograms were derived by direct sequencing of the RT-PCR products containing *ndhB* editing sites in WT, *ssa1*, Com1–3, *ssa1–2/1–9* seedlings. **B** RNA editing efficiency statistics of *ndhB* site in WT, *ssa1*, Com1–3, *ssa1–2/1–9* seedlings

PPR proteins have been reported to be involved in RNA editing [53]. We speculated that SSA1 was perhaps a new RNA editing factor. To detect whether *SSA1* is involved in RNA editing, we sequenced all 24 identified RNA editing sites in the chloroplast genome. The analysis results showed that *ndhB-737* had a 50% reduction in *ssa1*, whereas it was completely edited in WT (Fig. 6A-B). This editing event resulted in a Leu to Pro amino acid change at residue 246 of NADH dehydrogenase subunit 2 (*ndhB*) in WT. The other loci maintained similar editing efficiency between the WT and *ssa1* mutants (Fig. S9A-B). To understand the relationship between RNA editing and the genetic characteristics of *SSA1* in more detail, RNA editing tests were also performed on complementary lines and knockout lines. The results showed that complete recovery was not just phenotypic but also in RNA editing efficiency in Com1, Com2 and Com3. At the same time, the RNA editing efficiency of *ndhB-737* was greatly reduced in the *ssa1–2* and *ssa1–9* lines (Fig. 6A-B). These results suggest that SSA1 is not only involved in RNA splicing but also in RNA editing.

### SSA1 interacts with OsMORFs and OsTRXz

**Fig. 7:**
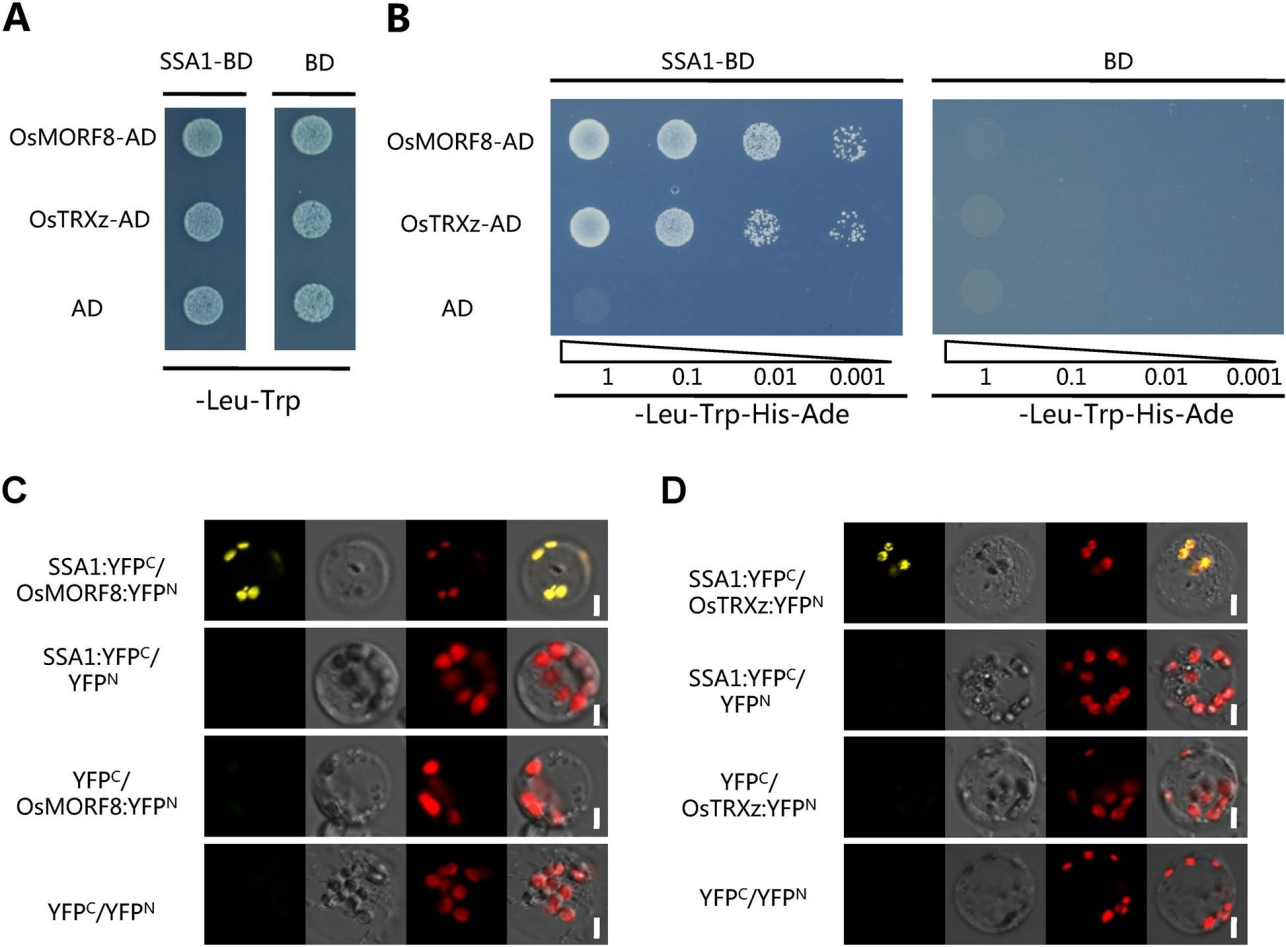
SSA1 physically interacts with OsMORF8 and OsTRXz. **A-B** Ayeast two-hybrid assay showing the interaction between SSA1 and MORF8/OsTRXz in -Trp-Leu/SD culture medium(**A**) and -Trp-Leu-His-Ade/SD culture medium (**B**). **C** A BiFC assay in rice protoplast showed the interaction between SSA1 and OsMORF8. Bar = 5 μm. **D** A Bifc assay in rice protoplast showed the interaction between SSA1 and OsTRXz. Bar = 5 μm

To further study the SSA1 polymorphism through the rice gene expression FREND network (https://ricefrend.dna.affrc.go.jp), we performed an enrichment analysis of the first 100 coexpressed genes of *SSA1* and found that *SSA1* was coexpressed with proteolytic enzymes and RNA processing-related genes. Considering that *SSA1* encodes the PPR protein and is coexpressed with RNA processing-related genes, we focused on candidate genes related to RNA processing. To accurately study the SSA1 polymorphism, we screened a rice yeast library. A clone encoding OsMORF8 in ricewas repeated at least three times. We further used a yeast two-hybrid experiment to verify the interaction between SSA1 and OsMORF8 in rice. The protein interactions can be repeated in double-transformed yeast (Fig. 7A-B). Meanwhile, SSA1 and OsMORF8 were cotransformed into rice protoplasts, and the BiFC experiment further verified that SSA1 could interact with OsMORF8 (Fig. 7C). Recently, OsTRXz was reported to interact with OsMORF8 [61, 65]. We tried to use Y2H and BiFC to determine the relationship between SSA1 and OsTrxZ. Both of the results showed that SSA1 could also interact with OsTRXz, which participates in RNA editing of chloroplast genes (Fig. 7A-B, D). Overall, the coexpression network of *SSA1* and the in vitro and in vivo assays verified that OsMORF8 and OsTRXz were the partners of SSA1 in RNA processing.

## Discussion

### Characteristics of *SSA1* in chloroplast development

The colour of plants and their leaves often indicates the chlorophyll content and chloroplast development status of plants. Common leaf colour-changing mutants include albino, chlorosis, light green, staygreen, zebra, and green-revertible in rice [11, 20]. According to Oryzabase (https://shigen.nig.ac.jp/rice/oryzabase/), at least 200 leaf colour changing mutants have been reported, and their candidate genes have been cloned in rice. These mutants provide important genetic resources for understanding the regulatory mechanisms of chlorophyll synthesis and chloroplast development [33, 35, 68]. In this study, we isolated a rice mutant, *ssa1*, which has an albino phenotype in plants, and used mapping to identify the target gene controlling this phenotype (Figs. 1A-D and 2A). In the *ssa1* mutant, malformation of chloroplasts was an internal factor that caused an albino phenotype in leaves (Fig. 1F-H). These macro and micro phenotypes are similar to those of the previously reported mutants *al1* and *asl2,* which encode the sole octotricopeptide repeat protein (RAP) and plastid ribosome proteins (PRPs) in rice, respectively [29, 80]. Both *AL1* and *ASL2* influence chloroplast synthesis by regulating the biological functions of ribosomes [29, 80]. In regard to *ssa1*, the phenotype was similar to that of *al1* and *asl2* but controlled by a PPR protein that is quite different from RAP and PRPs. In the process of studying *ssa1*, we found that ribosomal RNA in the chloroplast could not be synthesized normally (Fig. 5D-F). Later studies found that gene deletion of *SSA1* leads to abnormal RNA splicing and RNA editing. Considering that the genes involved in abnormal splicing are *ycf3* and *atpF* and *ndhB* is also involved in abnormal RNA editing, these genes are not directly related to ribosome formation and homeostasis. Therefore, we believe that in *ssa1*, abnormal chloroplast development may leads to abnormal ribosomal RNA formation in chloroplasts. These characteristics are different from the regulatory mechanism of chloroplast development of *AL1* and *ASL2* by influencing ribosomes. Thus, our study provides new insights into early chloroplast development in rice.

### SSA1 is essential for maintaining PEP activity in Rice

**Fig. 8:**
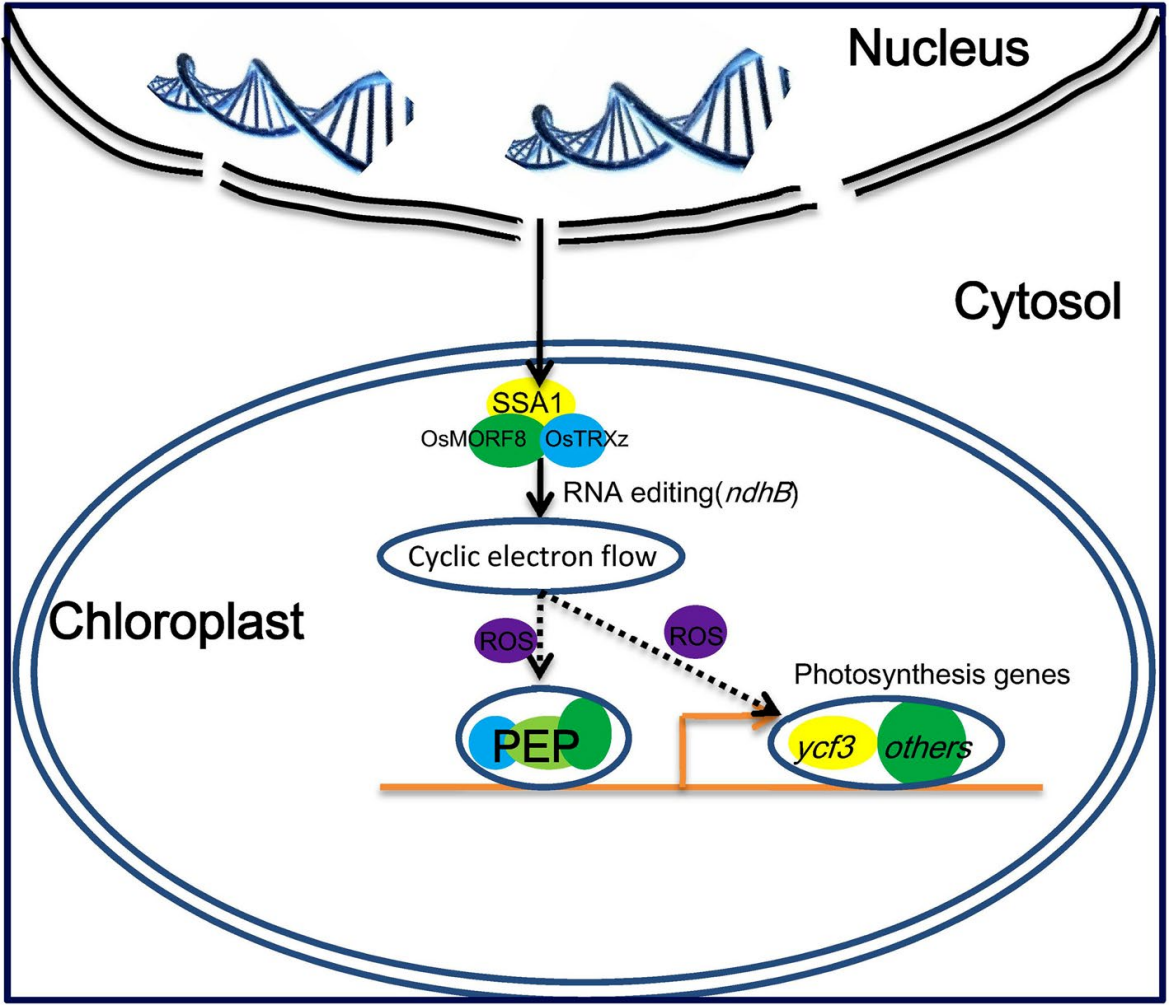
A schematic of possible mechanism of *SSA1* in RNA editing/splicing and chloroplast development. *SSA1* regulates RNA editing/splicing and is required for chloroplast development. SSA1 regulates RNA editing of some genes including *ndhB* (Circulating electron transport chain components in photosynthesis) along with OsTRXz and OsMORF8. The main characteristic of the *ssa1* mutant may its deficiency in RNA editing, and the remaining defects (e.g. chlorophyll content, gene expression, and ribosome biogenesis) are secondary. That *ndhB* cannot be edited normally may causes abnormal electron transport chain in *ssa1* chloroplast. Then, it may result in localized ROS accumulation in chloroplasts. The ROS may affect the PEP activity and gene expression including aberrant splicing *ycf3*

NEPs and PEPs play important roles in regulating chloroplast development. It has been reported that NEP transcribes the core components of the PEP complex, while PEP is known to specifically transcribe chloroplast genes, including *psbA*, *psaB*, and *rbcL,* that participate in photosynthesis and chloroplast development [28, 73]. Similar to the mutant *al1* and *dua1* at low temperature, the expression of most NEPs and PEPs was altered significantly [10, 80]. In our results, the expression level of four core genes of the PEP complex increased significantly (Fig. 5A), while the expression of photosynthetic electron transport chain- and photophosphorylation-related genes in chloroplasts decreased significantly in *ssa1* (Fig. 5C). Upregulated NEP-dependent core factor housekeeping genes (*rpoA*, *rpoB*, *rpoC1*, *rpoC2*) and downregulated expression of PEP-dependent photosynthesis genes (such as *psaB*, *psbA psbB* and *psbC*) are typical gene expression patterns due to impaired plastid transcription. Considering the results that there was a deficiency in RNA editing in *ndhB*, which plays roles in the electron transport chain, and locally elevated ROS (reactive oxygen species) in *otp51* [72], we hypothesized that the change in the expression level of NEP-dependent core factors and PEP-dependent photosynthesis genes indicated a response to abnormal accumulation of ROS (Fig. 8). These novel pathways affecting gene expression require further experimental validation in chloroplasts.

### SSA1 affects plastid group II intron splicing

RNA splicing is an important process of connecting exons to form a continuous mRNA molecule for translation [37]. PPR proteins play a very wide range of roles, including RNA stabilization, cleavage, splicing and editing [6]. Some mutants that are deficient in splicing *ycf3* have been reported before. In the dicotyledon plant *Arabidopsis thaliana*, the PPR proteins ACM1, PBF2, EMB1270 and ECD2 belong to the P-subfamily. Knockout of *ACM1* transgenic plants resulted in abnormal RNA splicing of *ndhA*, *ndhB* and *ycf3*. Meanwhile, the accumulation of chloroplast ribosomes was damaged, which led to albino cotyledons and seedling lethality [62–64]. PBF2 specifically plays a role in the splicing of *ycf3* intron 1 [61, 65], and EMB1270 interacts with CFM2 to facilitate the splicing of specific group II introns of *ndhA*, *ndhB*, *ycf3* and *clpP1* transcripts [77, 79]. ECD2 is also involved in the splicing of *ndhA*, *ycf3*, *rps12*, and *clpP1* transcripts in Arabidopsis chloroplasts [62–64]. Unlike PPRs in Arabidopsis, few P-type PPR proteins have been cloned in rice and their posttranscriptional regulatory mechanisms are not very clear. In *apo1* mutants, *ycf3–2* remains completely unspliced, and splicing defects result in the loss of photosynthetic complexes in Arabidopsis [66]. The PPR protein THA8 participates in many fields of organellar RNA metabolism and is associated with the splicing of group II introns of *ycf3–2* and *trnA*. When its function is inactivated, the plant shows a chloroplast malformation phenotype in maize [22]. All of these defective proteins display severe abnormal chloroplast development and a decreased chlorophyll content phenotype in plants. In our study, *SSA1* encoded a P-type PPR protein and was mainly expressed in young leaves (Fig. 4A-D). Further experimental results demonstrated that SSA1 participates in the splicing of *atpF* and *ycf3* during the early stage of plant development (Fig. S8). The plastid-encoded YCF3 protein contains three tetratricopeptide repeats (TPRs) that act as sites for protein–protein interactions for the PSI subunits in photosynthesis [42]. Thus, *ycf3* is essential for the accumulation of the photosystem I (PSI) complex and acts at the posttranslational level [42]. *OSOTP51*, which is homologous to *SSA1*, affects the intron splicing ofseveral plastid genes, including *ycf3*. The mutant showed albinism and abnormal chloroplast development in rice and Arabidopsis. By measuring chlorophyll fluorescence and studying the stability of the protein, it was found that photosystem assembly was abnormal when *OTP51*was deactivated [36, 53]. We obtained RNA splicing experiment results consistent with OSOTP51 and identified its interacting proteins. These data implied that*ycf3* is essential for the development of preplastids into plastids, including in the *ssa1* mutant.

### SSA1 facilitates RNA editing by forming an Editosome with OsMORF8 and OsTRXz

RNA editing is believed to be an important posttranscriptional regulatory mechanism for C-to-U RNA sequence alterations in plants. To date, hundreds of these sites have been found in chloroplasts and mitochondria [15]. MORFs and PPRs are essential for RNA editing at multiple sites. Recently, both WSP1/OsMORF2 and OsMORF9 were identified as multiple RNA editing factors in rice chloroplasts [74, 77, 79]. In addition, there is another MORF protein, OsMORF8, located in chloroplasts in rice [61, 65]. In *ssa1*, the RNA editing efficiency of *ndhB* transcription decreased significantly (Fig. 6A-B). We speculated that SSA1 and MORFs might cooperate in the chloroplast RNA editing process. Surprisingly, SSA1 did not interact with WSP1 and OsMORF9 but did interact with OsMORF8 (Fig. 7A-C). Moreover, we detected that OsTRXz, which is involved in *ndhB* RNA editing, also interacts with SSA1 [61, 65]. Furthermore, we found that *ndhB* RNA editing was reduced but not abolished in *ssa1* (Fig. 6A-B). We think that in addition to SSA1, other factors might participate in *ndhB* RNA editing in rice. Therefore, SSA1 may facilitate RNA editing by forming an editosome including OsMORF8, OsTRXz and other editing factors. The NAD(P)H dehydrogenase complex is thought to eliminate oxidative stresses in chloroplasts [60]. As an important component of the NDH complex, deficient *ndhB* RNA editing alters its activity or stability and eventually inhibits cyclic electron flow. Similar to *otp51*, it is speculated that a defective cyclic electron flow pathway could generate ROS in chloroplasts that may affect the function of the PEP in *ssa1* [72]. The elevated local ROS in chloroplasts may not affect the expression of nuclear genes. The increased expression of core factors (*rpoA*, *rpoB*, *rpoC1* and *rpoC2*) may be due to the negative feedback regulation of the nuclear gene *RpoTP*. As the *ndhB* gene is not edited normally in *ssa1*, nonfunctional PEP components indirectly influence *ycf3* splicing and PEP gene expression. The abnormal splicing of *ycf3* might be the main reason for the defective chloroplast biogenesis in *ssa1*, although this requires further experimental analyses.

## Conclusions

By screening photosynthesis-related mutants in our mutant library, we found the mutant *ssa1,* which has an albino phenotype at the seedling stage. Meanwhile, we explored the internal causes for the phenotype and found that the chloroplast of mutant *ssa1* was malformed and chlorophyll could not be synthesized normally. To further explore the molecular mechanism of this phenotype, we constructed a genetic population and carried out map-based cloning. The results identified a new allele of *OSOTP51* that encodes a P-type PPR protein that plays an important role in chloroplast development. The phenotype of the *ssa1* mutant exhibits albinism that results from malformed chloroplasts. Consistently, SSA1 deficiency caused abnormal RNA editing and splicing and impaired ribosome biosynthesis and PEP activity. Thus, our study clarified the internal correlation between RNA modification and chloroplast development.

## Materials and methods

### Plant material and growth conditions

The japonica variety Nipponbare has been conventionally cultivated in Japan. The seeds of Nipponbare were from WT plants affiliated with the author, and all of the mutant and transgenic plants in this study were based on the Nipponbare background. The *ssa1* mutant comes from the mutant library constructed by EMS mutagenesis. The plants in the field were grown at the Langfang Experimental base in Hebei Province. When in the incubator, the plant growth conditions were 28 °C and illumination period (light/dark 10/14), and the relative humidity was 60%. The plants used for mapping seedlings were grown in fields. The seedlings were grown in incubators for phenotypic observation and quantitative detection.

### Map-based cloning

The heterozygous *ssa1* mutant was hybridized with Dular, another indica rice ecotype, to determine its dominant or recessive relationship. The F2 generation was used as the genetic population for gene mapping. First, genomic DNA of 24 single plants with obvious phenotypes was extracted and used to construct four mapping mixed pools. The linkage between phenotypes and mutation sites was screened by using original molecular markers in our lab.The chromosome where the mutation sites were located was initially identified. Molecular markers were further designed near the mutation sites, and the number of individual plants was increased until the mutation locus was determined. PCR amplification and sequencing were performed on the genes in the interval to determine the specific mutation style of the target gene.

### Complementation of *ssa1* and knockout of *SSA1*

For complementation tests of the *ssa1* mutant, the CDS of *SSA1* was cloned into the pCAMBIA2300 binary vector and expressed by the driving actin promoter. CRISPR/Cas9 technology followed the guide of reference [40]. The speci [8] fic target sequences for *SSA1*were selected by using CRISPOR ( [12]; ). The target site was connectedto the U3 promoter and fused with gRNA before being loaded into the expression vector YLCRISPR/Cas9-MH. The complementary binary vector was transferred into *ssa1* mutant calli, while CRISPR/Cas9 was transferred into WT calli by the Agrobacterium-mediated method.

### Measurement of pigment contents

The leaves of WT, *ssa1* or transgenic seedlings were selected, and the same weight (M) was taken from each for chlorophyll extraction. The sample was added to a volume of 80% ethanol and extracted in a 70 °C water bath for 12 hours. After all the green on the leaves had faded, the absorbance values of the WT, *ssa1* and transgenic seedling chlorophyll extracts at 663 nm and 645 nm were measured and analysed by a colorimetric dish with an optical path of 1 cm.

### RNA isolation and qRT–PCR analyses

The RNA of various plant tissues was isolated bycolumn adsorption according to the kit protocol (QIAGEN, Cat# 74204). The RNA was reverse transcribed using PrimeScript II Reverse Transcriptase (TaKaRa) with oligo (dT) primers for nuclear-encoded genes or random primers for plastid-encoded genes. For spatiotemporal expression of *SSA1* and representative genes encoded by plastids, real-time quantitative PCR was conducted by using SYBR Premix as a fluorescence indicator on the BIO-RAD Real-Time PCR System. *Actin (Os01g0376700)* was used as a reference gene. Relative expression was calculated using the 2^−ΔΔCT^ method as described previously [21]. Each final value displayed is the average of the three physiological and three technical replicates. Specific primers for qRT–PCRare listed in Supplementary Table S1.

### Transmission Electron microscopy

The main TEM protocol was as follows [57]. The fresh rice leaves were cut into 0.5 cm × 0.5 cm pieces and immediately submerged in fixative solution (2% formaldehyde and 2% glutaraldehyde in 0.1 M Na-cacodylate buffer) followed by vacuum supply for 15 min to ensure that the sample was immersed in solution. Then, the fixed samples were washed and fixed in osmium tetroxide for 2 h followed by a series of different concentration gradients of ethanol. Absolute ethyl alcohol was replaced by anhydrous acetone before embedding inresin. After the embedding of the sample was complete, the polymerization was carried out at temperatures and times as follows: 37 °C, 12 h; 45 °C, 12 h; 60 °C, 12 h. After Epon polymerization, 75 nm thin sections were cut using a diamond knife and mounted on copper grids. Then, the sampleswere imaged using a JEOL JEM-1400 TEM at 80 kV equipped with a Gatan Ultrascan 1000 CCD camera.

### Analysis of RNA editing and splicing

For RNA editing, RNA was extracted from WT or *ssa1* plants and treated with DNA enzymes before reverse transcription to reduce the influence of genomic DNA on the experimental results. Primers were designed and amplified for 24 chloroplast genome editing sites that have been reported in rice chloroplasts [16]. For RNA splicing analysis, the selection of sites was based on the literature [55, 74] and ensured to have at least one intron in the chloroplast genes. The siteswere amplified by using RT–PCR with primers flanking the introns. The splicing effect was analysed by agarose gel electrophoresis.

### Subcellular localization and BiFC analyses

The CDS of *SSA1* was cloned into pAN580 by seamless cloning (TaKaRa) to generate 35S::SSA1-GFP. Chloroplast autofluorescence was used as a chloroplast localization marker. For bimolecular fluorescence complementation (BiFC), the SSA1 sequence was cloned into pSPYCE, and TRXz and OsMORF8 were cloned into pSPYNE [58] at the SpeI and ClaI restriction sites to generate the three transient expression vectors. Two-week-old WT plants were used to dissociate protoplasts, and pAN580 was single transferred for subcellular localization. The BiFC vector pSPYNE-TRXz/OsMORF8 was cotransferred with pSPYCE-SSA1. The fluorescence signal was observed by confocal microscopy (Zeiss, LSM700) after incubation at room temperature in the dark for 16 hours.

### GUS staining analysis

The binary expression vector SSA1::GUS in pCAMBIA1391Z was transformed into WT plants. At least 20 independently generated transgenic lines were assayed for promoter expression pattern analysis. First, the vector with the frame of pCAMBIA1391Z was selected, and the promoter sequence of *SSA1*was amplified to drive GUS. The primers for amplification are shown in Table S1. The construct was used to infect rice calli in the presence of *Agrobacterium tumefaciens*. The resistant callus was screened, and the transgenic seedlings were identified. The fresh sample tissue that needed to be tested was immersed into staining solution (0.1 M PBS; 10 mM EDTA; 2 mM K3Fe(CN)6; 2 mM MK_4_Fe(CN)_6_; 0.1% Triton X-100; 1 mM X-gluc), and vacuum was supplied to force the solution to invade the tissue. The staining was observed frequently and photographed.

### Yeast two-hybrid analysis (Y2H)

The CDSs of *SSA1*, *OsTRXz*, and *OsMORF8*were amplified and inserted into the bait plasmid pGBKT7 and the prey plasmid pGADT7 between the restriction enzyme cutting sites of EcoRI and BamHI. The positive clone was tested by bacterial colony PCR and sequencing. The bait and prey plasmids were cotransformed into yeast strain AH109 by PEG-mediated spreading of SD/−Trp/−Leu medium and SD/−Trp/−Leu/−His/−Ade medium. The target yeast colony was cultured at 28 °C for 3 days. The primer sequences are listed in Supplemental Table S1.

### Sequence analyses and phylogenetic studies

To identify homologues of *SSA1*, we used full-length SSA1 amino acids to search homologous sequences from other species by NCBI (https://www.ncbi.nlm.nih.gov/). We selected some of the more homologous species shown in NCBI to perform sequence alignment analysis using DNAman and build a phylogenetic tree using the maximum likelihood method usingMEGA version 6 [14]. Default settings were used.

## Supplementary Information

**Additional file 1: Fig. S1.** Mutational analysis of *ssa1*. **Fig. S2.** Construction of complementary vectors and detection of chlorophyll content. **Fig. S3.** Amino acid sequence alignment of the WT and knockout lines *ssa1–2/1–9*. **Fig. S4.** Sequence analysis of SSA1. **Fig. S5.** Sequence alignment of PPR protein with sequence homology to SSA1 in rice. **Fig. S6.** Phylogenetic tree showing predicted relationships between SSA1 and other closely related species. **Fig. S7.** Expression analysis of *SSA1* in different rice tissues. **Fig. S8.** Splicing analyses of rice chloroplast transcripts in WT and *ssa1* mutant. **Fig. S9.** RNA editing efficiency of various target sites.

**Additional file 2: Table S1.** Primers used in this study.

## Data Availability

The datasets supporting the results of this article are available from thecorresponding author upon reasonable request. Sequence data used duringthe current study for the cDNA and genomic DNA of SSA1, OsMORF8 and OsTRXz are available from the GenBank data libraries under accession numbersLOC_Os02g47360 (http://rice.uga.edu/cgi-bin/sequence_display.cgi?orf=LOC_Os02g47360.1), LOC_Os09g33480 (http://rice.uga.edu/cgi-bin/sequence_display.cgi?orf=LOC_Os09g33480) andLOC_Os08g29110 (http://rice.uga.edu/cgi-bin/sequence_display.cgi?orf=LOC_Os08g29110),respectively, and could also be availablefrom the National Center for Biotechnology Information (NCBI).*SSA1*Gene ID: LOC4330440 (https://www.ncbi.nlm.nih.gov/nuccore/NC_029257.1?report=genbank&from=28917374&to=28921066), OsMORF8 Gene ID: LOC4347529 (https://www.ncbi.nlm.nih.gov/nuccore/NC_029264.1?report=genbank&from=19737162&to=19740278&strand=true), OsTRXz Gene ID: LOC4345435 (https://www.ncbi.nlm.nih.gov/nuccore/NC_029263.1?report=genbank&from=17818423&to=17820524&strand=true).

## Abbreviations

*ssa1*: *Seedling stage albino1*
WT: Wild type
TEM: Transmission electron microscope
PPR: Pentapeptide repeat protein
GFP: Greenfluorescent protein
YFP: Yellow fluorescent protein
NEP: Nuclear-encoded RNA polymerase
PEP: Plastid-encoded RNA polymerase
BiFC: Bimolecular fluorescence complementation
Y2H: Yeast Two-Hybrid
