# Supplementary material for: A chloroplast-localized pentatricopeptide repeat protein involved in RNA editing and splicing and its effects on chloroplast development in rice
Source: BMC Plant Biol. 2022 Sep 13;22:437. doi: 10.1186/s12870-022-03819-y (PMC9469629; doi:10.1186/s12870-022-03819-y)
Supplement: Supplementary file 1 — Additional file 1: Fig. S1. Mutational analysis of ssa1. Fig. S2. Construction of complementary vectors and detection of chlorophyll content. Fig. S3. Amino acid sequence alignment of the WT and knockout lines ssa1–2/1–9. Fig. S4. Sequence analysis of SSA1. Fig. S5. Sequence alignment of PPR protein with sequence homology to SSA1 in rice. Fig. S6. Phylogenetic tree showing predicted relationships between SSA1 and other closely related species. Fig. S7. Expression analysis of SSA1 in different rice tissues. Fig. S8. Splicing analyses of rice chloroplast transcripts in WT and ssa1 mutant. Fig. S9. RNA editing efficiency of various target sites. [file 12870_2022_3819_MOESM1_ESM.docx]

**
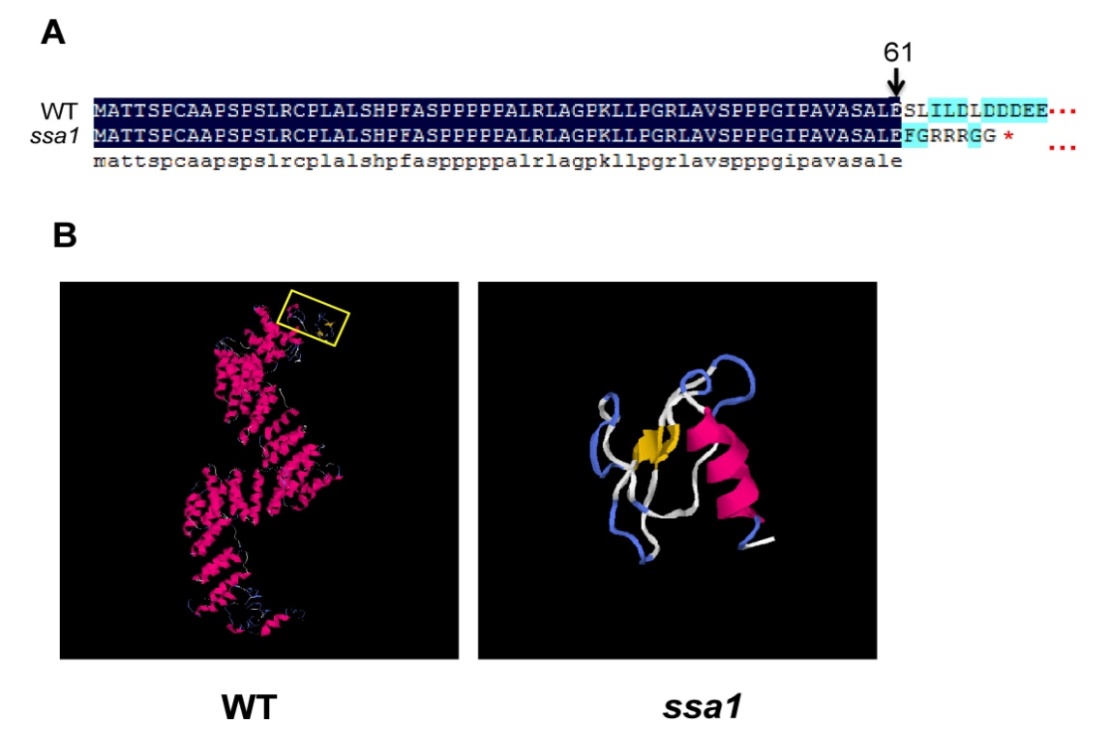
Fig. S1 The mutational analysis of *ssa1***.


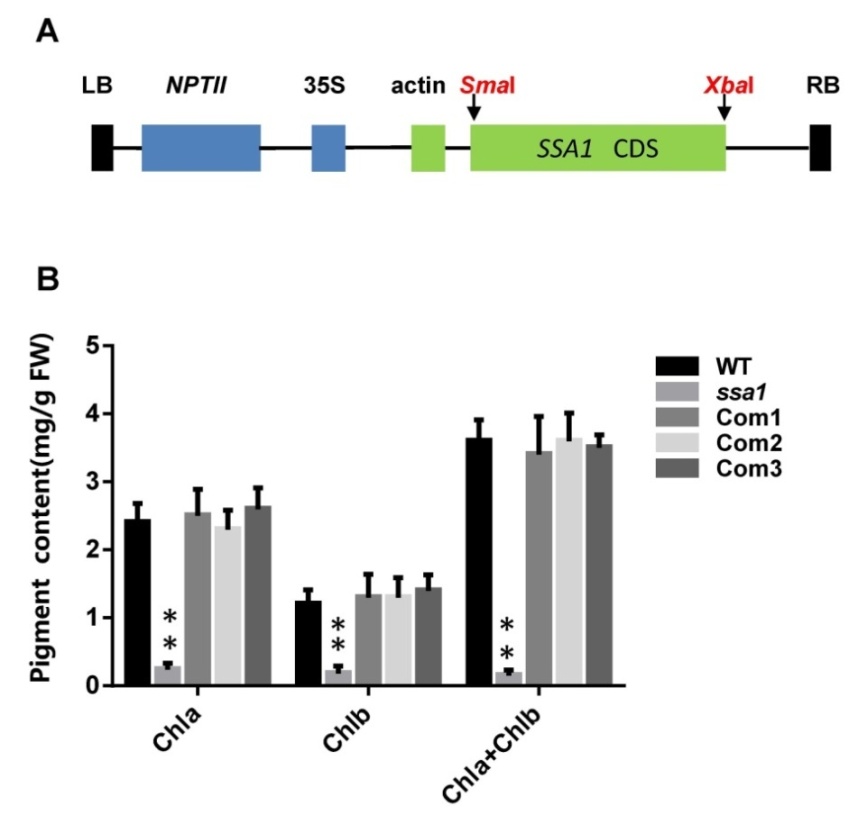
(A) Amino acid sequence alignment of the WT and *ssa1* mutant. (B) Deletion of functional domains shown by modeling the three dimensional structures of SSA1 and the truncated *ssa1* proteins.

**Fig. S2 Construction of complementary vectors and detection of chlorophyll content.**

(A) Schematic diagram of complementary carrier. The full length CDS of *SSA1* was amplified and

inserted behind the *actin* promoter. (B) Chlorophyll a and chlorophyll b content detection in WT, *ssa1* and Com1-3. SD was calculated from 5 independent plant. (**P<0.01,Student’s t test)

**
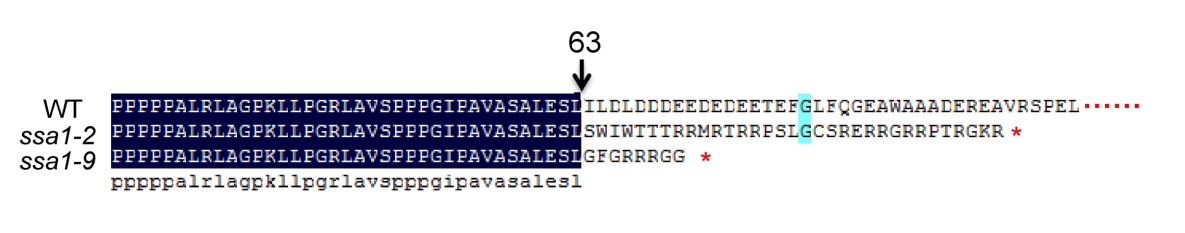
Fig. S3 Amino acid sequence alignment of the WT and knockout line *ssa1-2/1-9*.**

**
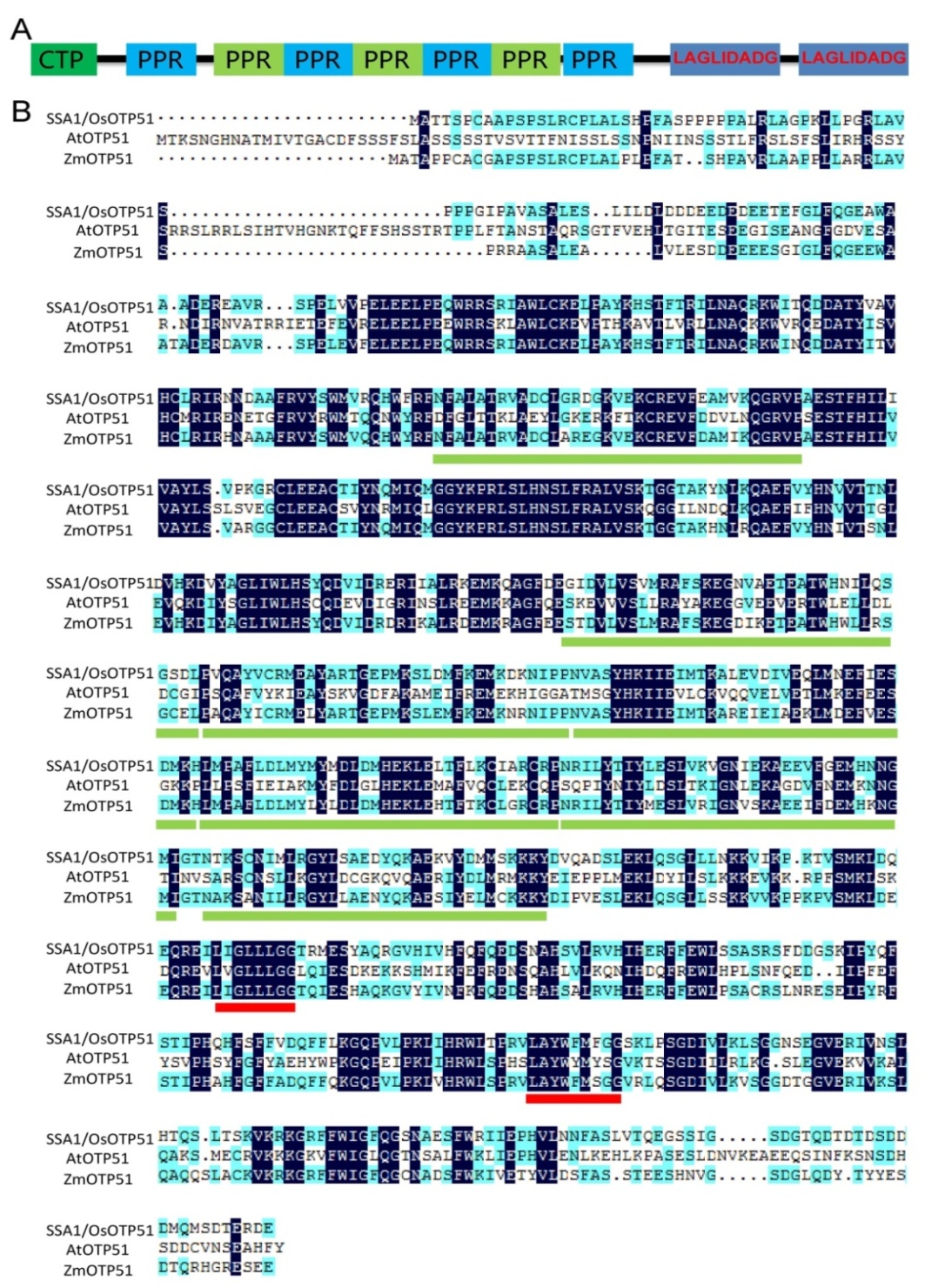
Fig. S4 The sequence analysis of SSA1**

(A) The predicted SSA1 protein motif. (B) Multiple sequence alignments of SSA1 amino acide and its homologs. Oryza sativa (*LOC_Os02g47360*, *SSA1/OsOTP51*), Arabidopsis thaliana (*AT2G15820*), and Zea mays (*GRMZM2G028605*). Conserved residues are shaded in dark blue , identical residues in binght blue. Each PPR repeat is indicated with green. The specific amino acids for PPR protein RNA specific interaction are indicated by black arrows.

**
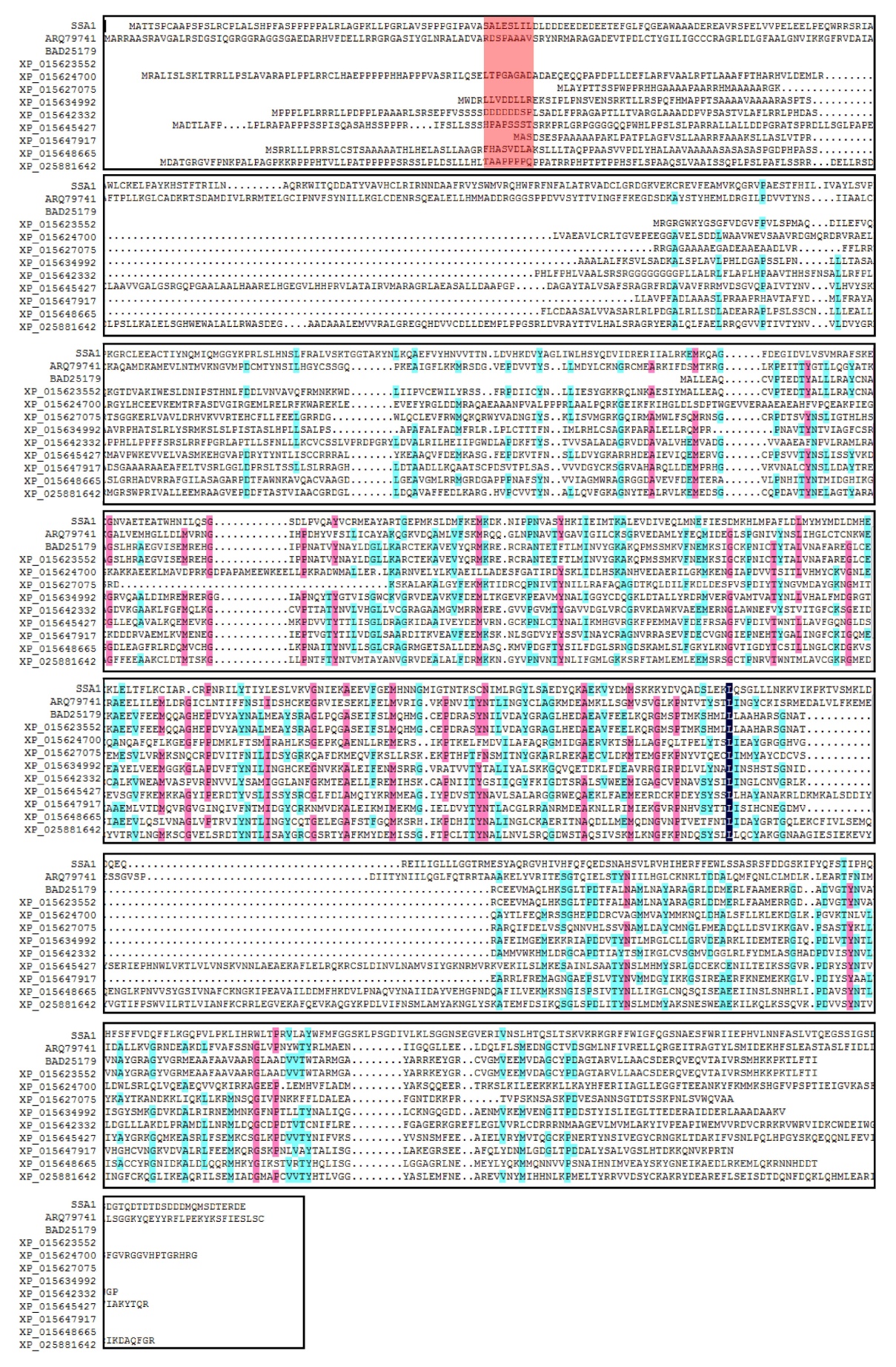
Fig. S5 Sequence alignment of PPR protein with sequence homology to SSA1 in rice**

**The sequence were searched from NCBI. The red shading indicates the target site of the CRISPR-Cas9 knockout**

**
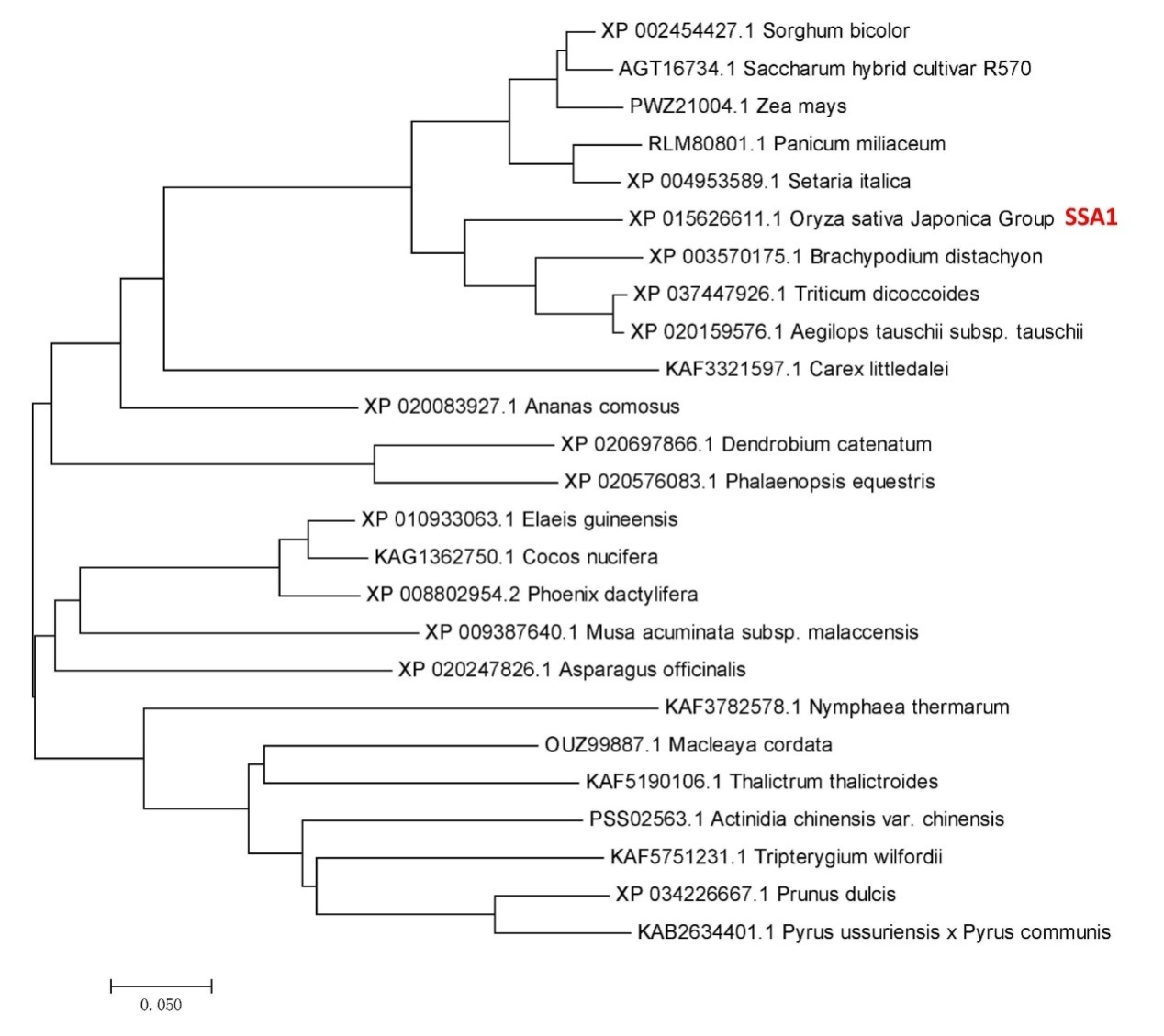
**

**Fig. S6 Phylogenetic Tree Showing Predicted Relationships between *SSA1* and other closely related species**

**
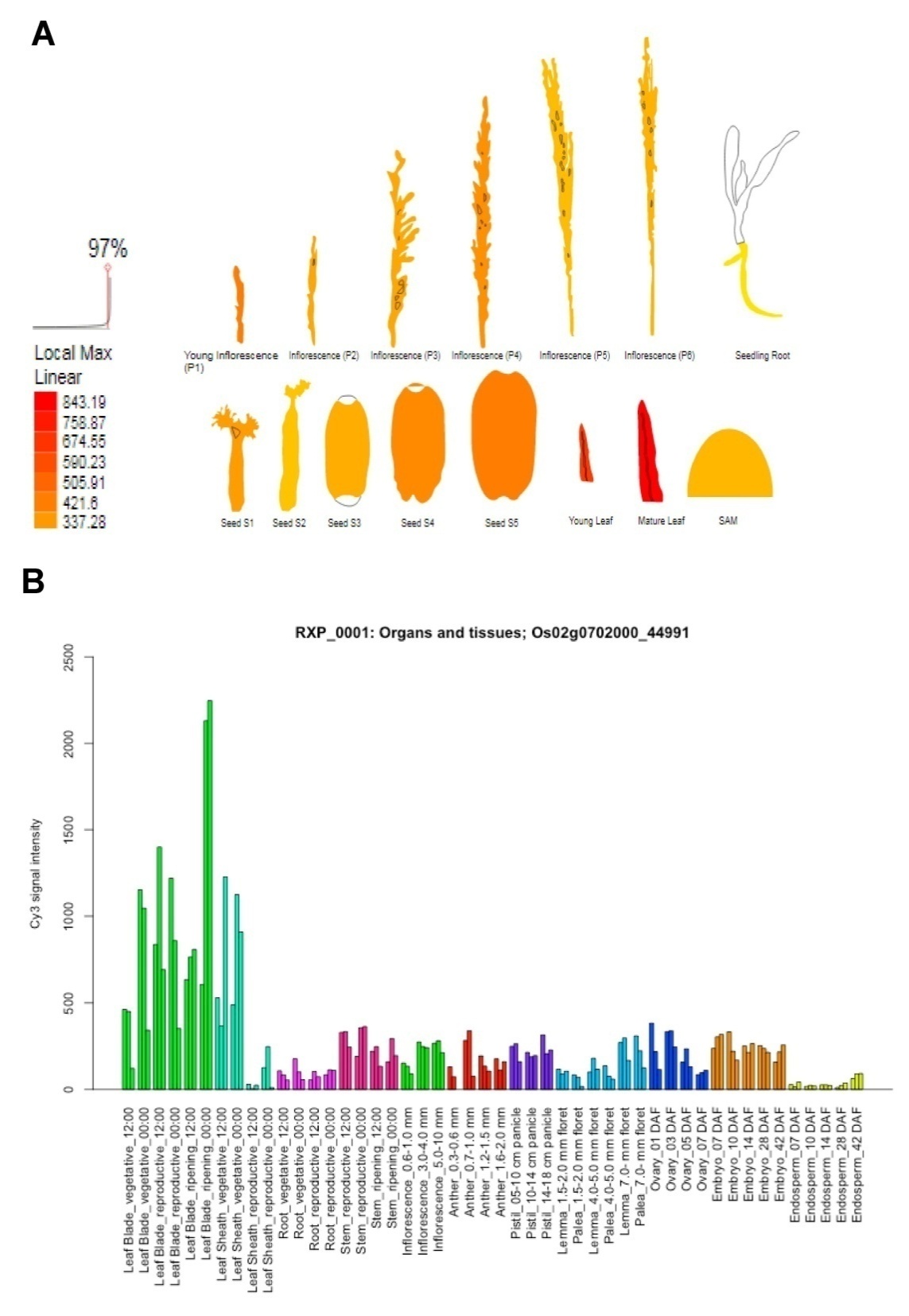
Fig. S7 Expression analysis of *SSA1* in different rice tissues*.***

(A) Expression profiles of *SSA1* gene from http://bar.utoronto.ca/eplant_rice/#

(B) Expression profiles of *SSA1* gene from http://ricexpro.dna.affrc.go.jp/

**
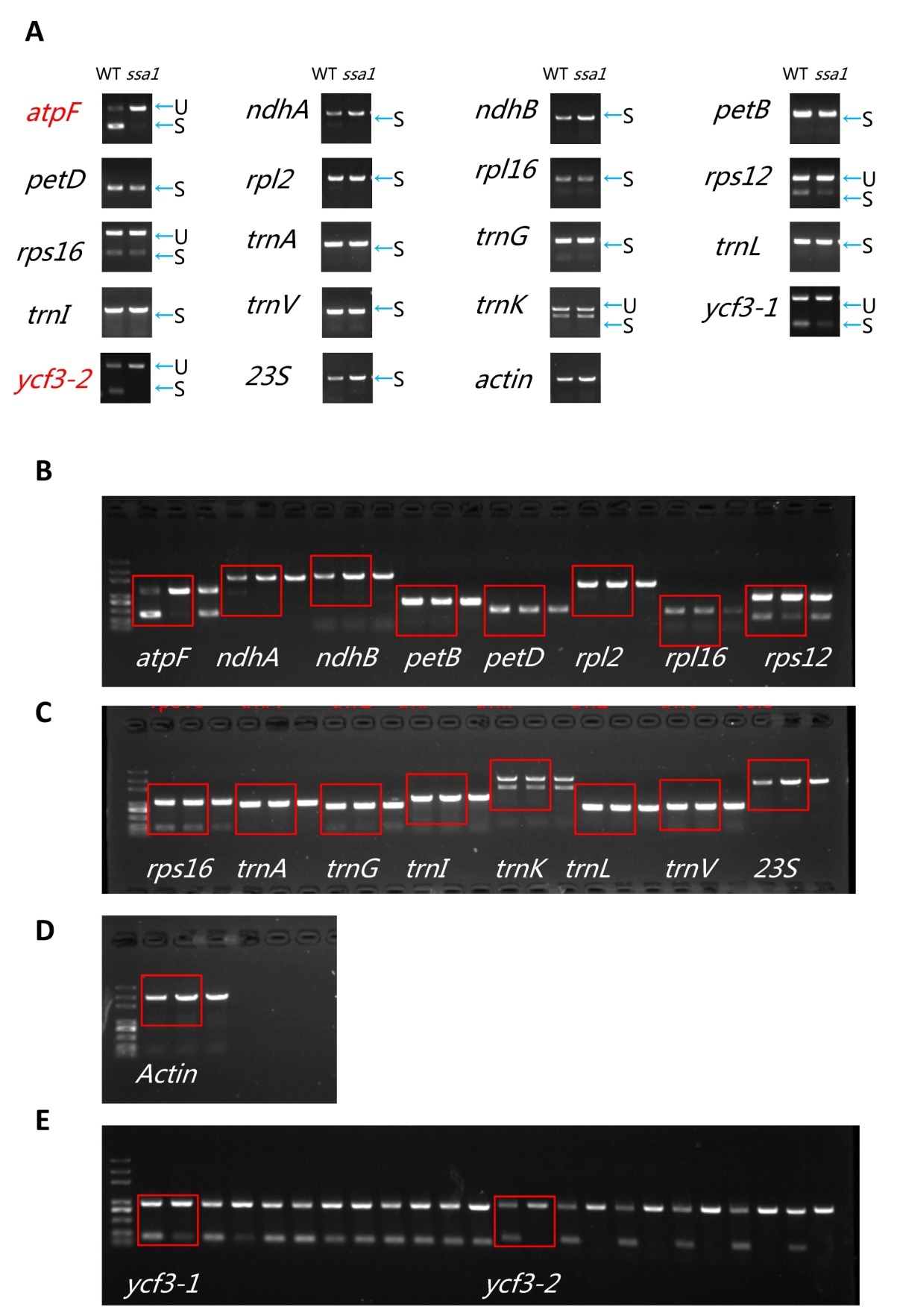
Fig. S8 Splicing analyses of rice chloroplast transcripts in WT and *ssa1* mutant.**

(A) Gene transcripts are labeled at the left. Spliced (S) and unspliced (U) transcripts are shown at the right. RNA was extracted from WT and *ssa1* mutant plants. The gels cropped from different parts of the several gels that in supplement materials. (B-E) The image before cropping in Figure (A).


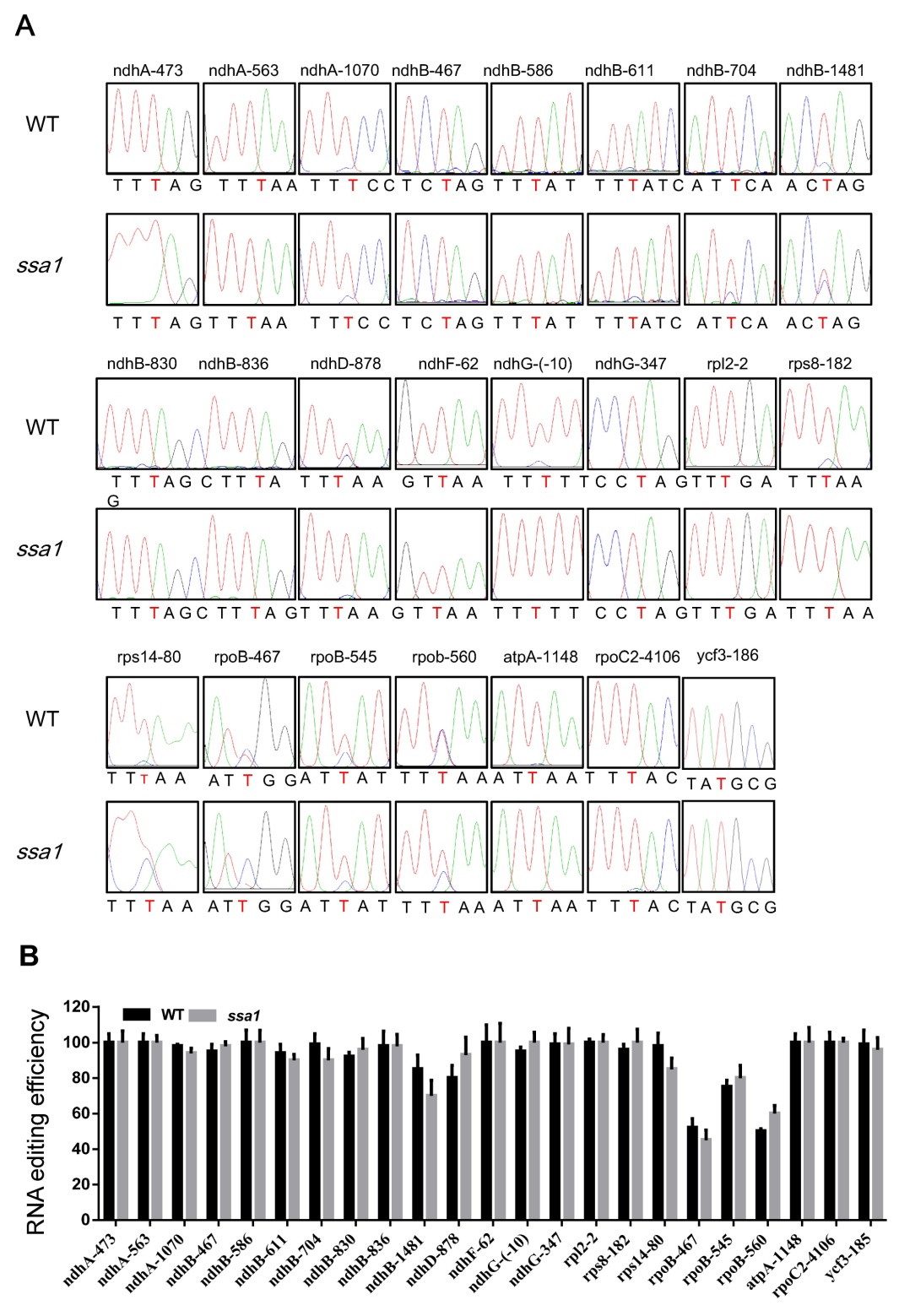
**Fig. S9 RNA editing efficiency of various target sites**

(A) Sequencing chromatograms showed the RNA editing state of plasid sites between WT and *ssa1*. (B) Editing efficiency of 24 plastid RNA editing sites in rice (*Oryza sativa)* WT and *ssa1* mutant plants. Error bars indicate SD (n = 3).
