# Supplementary material for: A chloroplast-localized pentatricopeptide repeat protein involved in RNA editing and splicing and its effects on chloroplast development in rice
Source: BMC Plant Biol. 2022 Sep 13;22:437. doi: 10.1186/s12870-022-03819-y (PMC9469629; doi:10.1186/s12870-022-03819-y)
Supplement: Supplementary file 2 — Additional file 2: Table S1. Primers used in this study. [file 12870_2022_3819_MOESM2_ESM.doc]

**Table S1–** Primers used in this study

| **Primers** | | **SEQUENCE（5'-3'）** |
| --- | --- | --- |
| **For Mapping** | | |
| rj2792F | AACTTGCAGGCAGAATAGCTG | |
| rj2792R | GCGGCTCTCAATTGTTGAA | |
| rj317F | TAAAATTGGCCTTCGCTTGT | |
| rj317R | TGCGAATATTTGGGGTCCTA | |
| rj2805F | TGTTACTCACTCCGTCCTAAAATG | |
| rj2805R | AACGGCTCGTTTGTAGGGTG | |
| InD2872F | GAGGTAAGAGCAAGTTTAATGGT | |
| InD2872R | CTCACCTAGCATATACTTAGCCAA | |
| InD2891F | TCACCTGAACAGGATAATCAAAATG | |
| InD2891R | ATTGACAGATTCGATCCATAGCTA | |
| InD2922F | TTCATCTAGCAGCACGCGAA | |
| InD2922R | AACCTACTGGGCTCTAGCGC | |
| InD2952F | ACCCTAGTTTCCGTACCCAATAT | |
| InD2952R | TACGGTTGGGCTCGTCTCG | |
| **For Mutant Sequencing** | | |
| *ssa1-F* | TATCCAAATCTCACAGGAGTGG | |
| *ssa1*-R | TCGTCCTGGGTGATCCACTT | |
| **For qRT-PCR** | | |
| ssa1-qPCR-f | GATGATAGGTACTAACACGAAGTCG | |
| ssa1-qPCR-f | CCATTCGAGTGCCTCCCAGA | |
| atpA-F | TCGCGGAGTTACAAGCCTTT | |
| atpA-R | ATTCTCGTAATCGTCGGCCC | |
| atpB-F | GGGGGAGCTGGAGTAGGTAA | |
| atpB-R | CCCCTACTCCGCCAAATACG | |
| atpI-F | ATTAGTCGTTGTTGTTCTTGT | |
| atpI-r | GCTTGAATACCGCTTGTAA | |
| petB-F | ACGTGGAAGTGCTAGTGTGG | |
| petB-R | CGGCAGTAAGCAGAGGAAGT | |
| petD-F | CCGACGGGCTTATTGACAGT | |
| petD-R | GGTTGTAGCTACTGGGCGAC | |
| psaA-F | GCAGTCGGATGTTTGGGGTA | |
| psaA-R | GAAATCTCGGAGCCACCCAT | |
| psaB-F | ATGAACGGACACCTTTGGCT | |
| psaB-R | CCCACCAATCTTGCTTGCAC | |
| psbA-F | TGTGGCCGCTCATGGTTATT | |
| psbA-R | CCCTACTACAGGCCAAGCAG | |
| psbB-F | GTTGAACAAGTAGGCGTAA | |
| psbB-R | ACAGTAGCAGGATCAGAATA | |
| psbC-F | GCTCCTTTAGGCTCTTTA | |
| psbC-R | GTCGCTAACCAACTTCTA | |
| psbD-F | GGCCCAATCGCTGTTTTTGT | |
| psbD-R | CCAAAACTCGGCGCAAAGAA | |
| rbcL-F | AGGGGAACGCGAAATGACTT | |
| rbcL-R | AAAGATACCGCGAGCACGAT | |
| rpoA-F | CCGCCTTCCGTAGAAATCGT | |
| rpoA-R | CGCGATCTCTCTTGATCCGT | |
| rpoB-F | GGCAGAAGAACTTGAGAA | |
| rpoB-R | GGAAGAATAGGTGAGTGATT | |
| rpoC1-F | CGTGCTACTTCTAATGTT | |
| rpoC1-R | CCTGTATGCCTAATCTATG | |
| rpoC2-F | GGCTGATGATGAGGTAAG | |
| rpoC2-R | TGAAGAATAGAAGGATAGACAA | |
| HEMA1-F | CGCTATTTCTGATGCTATGGGT | |
| HEMA1-R | TCTTGGGTGATGATTGTTTGG | |
| CHLD-F | GCTTGCAGAAAGCTACACAAGC | |
| CHLD-R | AGGCCGTGAGCTAAAGGAGC | |
| YGL1-F | ATGAACCTTACCGTCCTATTCCTT | |
| YGL1-R | GGACCCACCAACAGCAAGAT | |
| PORA-F | TGTACTGGAGCTGGAACAACAA | |
| PORA-R | GAGCACAGCAAAATCCTAGACG | |
| CAO-F | GATCCATACCCGATCGACAT | |
| CAO-R | CGAGAGACATCCGGTAGAGC | |
| DVR-F | CGAGCCCAGGTTCATCAAGGTGC | |
| DVR-R | CCTCCCGATCTTGCCGAACTCC | |
| ACTIN-F | TGACGGAGCGTGGTTACTCATTCA | |
| ACTIN-R | TCTTGGCAGTCTCCATTTCCTGGT | |
| **For Subcellular Localization** | | |
| SSA1-pAN580-F | GGAGCTAGCTCTAGAATGGCCACCACCTCTCCCT | |
| SSA1-pAN580-R | GCTCACCATGGATCCCTCGTCTCTTTCTGTATCGGACA | |
| **For Complementation Construct** | | |
| SSA1-23A-F | GAAGAGGTACCCGGGATGGCCACCACCTCTCCCT | |
| SSA1-23A-R | CAGGTCGACTCTAGATCACTCGTCTCTTTCTGTATCGG | |
| **For** **Complementation background test** | | |
| SSA1-background-f | ATGCCCTCTCGCGCTCTC | |
| SSA1-background-r | AGAGCGTGTGAAGGCGTGTA | |
| **For** **Complementation molecular identification** | | |
| SSA1-transf-F | ATGCCCTCTCGCGCTCTC | |
| SSA1-transf-R | CCTGGGTGATCCACTTGCG | |
| **Yeast two-hybridization** | | |
| SSA1-BD-F | ATGGAGGCCGAATTCATGGCCACCACCTCTCCCT | |
| SSA1-BD-R | CAGGTCGACGGATCCTCACTCGTCTCTTTCTGTATCGG | |
| MORF8-AD-F | GAGGCCAGTGAATTCATGGCGTCGGCGTCGC | |
| MORF-AD-R | GAGCTCGATGGATCCCTACTGGTAATTCCTCCCTTGTCC | |
| Trxz-AD-F | GAGGCCAGTGAATTCATGGCCATGGCCGCGG | |
| Trxz-AD-R | GAGCTCGATGGATCCTCACAATTCATTATCAATGATATTTCTG | |
| **For GUS construction** | | |
| SSA1-GUS-F | GACCATGATTACGCCAAGCTTCTCTGAATATATTGGTTGCCCTAT | |
| SSA1-GUS-R | CCAGTGAATTCCCGGGGATCCGGAGGATTCGACCGCGTA | |
| **Primers for RNA editing** | | |
| ndhA1-F | GGACCGTCTATAGCAGTCAT | |
| ndhA1-R | CTGACGCCAAAGATTCCATC | |
| ndhA2-F | GGTGGAATTTGTCTATTCCC | |
| ndhA2-R | TTTCTCTTGTTTGAGAGGAC | |
| ndhB1-F | GGCTATAACAGAGTTTCTGT | |
| ndhB1-R | CCAGAAGAAGATGCCATTCG | |
| ndhD-F | GGTTACCAGATACCCATGGG | |
| ndhD-R | AAAATAGCTCCATTGAGTCC | |
| ndhF-F | CAATATGCATGGGTAATCCC | |
| ndhF-R | AACCAGGATTCCTACAGTAG | |
| ndhG-F | CCTAATCCCTTTTTTCTTCC | |
| ndhG-R | TCAAGACATTTATAGCTCCC | |
| Rpl2-F | CCGGGTTATTCTATTCCACT | |
| Rpl2-R | TACGCATTTCGATTAGGGTC | |
| Rps8-F | GGAACTGTTCGGGTAGTATC | |
| Rps8-R | GGAATTCCTTGATAGTTGGC | |
| rps14-F | GTTTGATTCAGAGAGAGAGG | |
| rps14-R | TTCTCGAAGTATGTGTCCGG | |
| rpoB-F | GTCCTGGTATTTACTACCGC | |
| rpoB-R | TCCCCACCTACACAAGCAAA | |
| atpA-F | GACTCAATCTGGAGACGTTT | |
| atpA-R | TAGTAGCTATCTGCTCTTCC | |
| Ycf3-F | TGTGGTAAGAAGGGGTTTCG | |
| Ycf3-R | GCGAATAATTCCGACAACCT | |
| rpoC-F | GGTCCTTGGGGATTCTTGAT | |
| rpoC-R | TCTTGTTTTGTGGGTAACGG | |
| **Primers for RNA splicing** | | |
| atpF-F | TTTTAGCTCACTGGCCATCC | |
| atpF-R | TTCATCGCCCTTTGTTTTTC | |
| ndhA-F | ATGATAATAGACAGGGTACAGG | |
| ndhA-R | TTATAGTGAAACAAGTTGGGAAG | |
| ndhB-F | ATGATCTGGCATGTACAGAATG | |
| ndhB-R | CTAAAAGAGGGTATCCTGAGCA | |
| petB-F | TTCTCATATACGGTTCTCGG | |
| petB-R | TAAAGGGCCCGAAATACCTT | |
| petD-F | ATGGGAGTAACAAAGAAACC | |
| petD-R | TGTTGCTCCAATACCTAACC | |
| rpl2-F | ACGGCGAAACATTTATACAA | |
| rpl2-R | TTACTTACGGCGACGAAGAATA | |
| rpl16-F | ATGCTTAGTCCCAAAAGAAC | |
| rpl16-R | AACCGAAGAAATTGACTTCG | |
| rps12-F | ACTATCAACCCCAAAAAACC | |
| rps12-R | TTTGGCTTTTTGACCCCAT | |
| rps16-F | AAAACGATGTGGTAGAAAGC | |
| rps16-R | AGAATTCCGCCTTCCTTAAA | |
| trnA-F | GGGGATATAGCTCAGTTGGT | |
| trnA-R | TGGAGATAAGCGGACTCGAA | |
| trnG-F | TCGTTAGCTTGGAAGGCTAG | |
| trnG-R | GCGGGTATAGTTTAGTGGTA | |
| trnI-F | TGGGCCATCCTGGACTTGA | |
| trnI-R | AGCTCAGTGGTAGAGCGCG | |
| trnK-F | GGTTGCCCGGGACTCGAA | |
| trnK-R | GGGTTGCTAACTCAATGGTAGAG | |
| trnL-F | GGATATGGCGAAATCGGTA | |
| trnL-R | TGGGGATAGAGGGACTTGA | |
| trnV-F | TAGGGCTATACGGATTCGAA | |
| trnV-R | AGGGCTATAGCTCAGTTCGG | |
| ycf3-F | ATGCCTAGATCCCGTATAAATG | |
| ycf3-R | TTATTCAAATTCAAAGCGCTTC | |
| 23S-F | TTCAAAAGAGGAAAGGCTTG | |
| 23S-R | AGAGAGCACTCATCTTGGGG | |
